# Snake fangs: 3D morphological and mechanical analysis by microCT, simulation, and physical compression testing

**DOI:** 10.1093/gigascience/gix126

**Published:** 2017-12-15

**Authors:** Anton du Plessis, Chris Broeckhoven, Stephan G le Roux

**Affiliations:** 1 CT Scanner Facility, Stellenbosch University, Stellenbosch, Private bag X1, South Africa, 7602; 2 Department of Botany and Zoology, Stellenbosch University, Stellenbosch, Private bag X1, South Africa, 7602

## Abstract

This Data Note provides data from an experimental campaign to analyse the detailed internal and external morphology and mechanical properties of venomous snake fangs. The aim of the experimental campaign was to investigate the evolutionary development of 3 fang phenotypes and investigate their mechanical behaviour. The study involved the use of load simulations to compare maximum Von Mises stress values when a load is applied to the tip of the fang. The conclusions of this study have been published elsewhere, but in this data note we extend the analysis, providing morphological comparisons including details such as curvature comparisons, thickness, etc. Physical compression results of individual fangs, though reported in the original paper, were also extended here by calculating the effective elastic modulus of the entire snake fang structure including internal cavities for the first time. This elastic modulus of the entire fang is significantly lower than the locally measured values previously reported from indentation experiments, highlighting the possibility that the elastic modulus is higher on the surface than in the rest of the material. The micro–computed tomography (microCT) data are presented both in image stacks and in the form of STL files, which simplifies the handling of the data and allows its re-use for future morphological studies. These fangs might also serve as bio-inspiration for future hypodermic needles.

## Introduction

The fangs of venomous snakes are highly modified for piercing the skin and ejecting venom into prey, providing venomous snakes with a significant evolutionary and ecological advantage. Snake fangs vary considerably in size and shape, and this morphological variation can be attributed to differences in body size, diet, and feeding behaviour. In advanced snakes, 3 types of venom-conducting fangs can be found: (1) closed fangs with an enclosed venom-conducting canal and suture line on the top surface where the 2 sides seem to close up, (2) entirely fused fangs with an enclosed venom-conducting canal, and (3) open groove fangs with venom ejected along the groove surface due to high viscosity of the venom.

In a recent experimental microCT campaign, we conducted a phylogenetically informed analysis of fang phenotypes [1]. By using static load simulations applied to the micro–computed tomography (microCT) data of each fang, we found that, despite differences in shape and size, stress distributions after applying a load were similar between the 3 fang phenotypes. The results of the study suggest that fangs might be biomechanically optimized. This Data Note is meant to highlight this exceptional dataset, providing details on the analysis and providing additional results not included in the original paper. This includes advanced morphological comparisons, more detailed load simulation results and physical compression test data, and extraction of elastic modulus values. The fang models used for simulations and for morphological measurements are included in the form of image stacks and segmented STL (sterolithography) files. These STL files are significantly smaller than full microCT datasets and provide dimensionally accurate 3D models of the fangs. This simplified format hopefully allows a wider usage of the dataset by other researchers.

## Materials and Methods

High-resolution x-ray CT scans were recorded at the Stellenbosch University CT facility [2], using optimized parameters for highest-quality scanning using nanoCT [3]. Voxel sizes were between 1 and 8 μm depending on fang size. Each fang was individually loaded in rigid foam in a vertical orientation, with the foam attached to a glass rod. Scan settings included 60 kV and 240 μA with the fast-scan option, resulting in ∼1 hour per sample scan time. Datasets were processed in VGStudioMax 3.0, and static load simulations were performed using the *Structural Mechanics Simulation* module. This module makes use of voxel-based load simulation, similar to finite element modelling, but without the need for meshing of surfaces. The simulation requires a binary segmentation in the form of a surface determination but does not require a mesh. Nevertheless, a mesh is also generated for simple data handling and can in principle also be used for simulation, with appropriate remeshing or creation of artificial voxel data based on the mesh file—this is discussed in more detail in the Supplementary Material. In this work, a nominal load of 5 N was applied to the tip of the fang (in a region covering roughly half the distance to the venom canal exit orifice) and applied along the direction of the tip. For this, 2 regions of interest (ROIs) were defined, 1 at the base, which is the fixed ROI, and 1 at the tip as described above, covering half the distance from tip to venom exit orifice. The region between venom exit orifice and tip was used as the reference to align the axes, for applying a load in plane (directly parallel to the tip region). Young's modulus values were taken from the literature as 20 GPa [4] and Poisson's ratio 0.3. The fang was held at its base and the load applied, with other parameters based on a compromise between simulation time and convergence of the simulation result to a low error value. In this series of simulations, the number of iterations used was 2000, with a simulation cell size equal to 4. The resulting Von Mises stress distributions could be analysed visually and quantitatively using in this case a 10% maximum interval from the statistical stress results. This means that local maxima (such as stress hotspots in sharp points) are effectively smoothed out and an average is found for each fang, irrespective of individual stress concentration regions. The idea is to find average stress values, depending on the bulk morphology of each fang. This method has recently been applied successfully in a study of tensile stresses around defects inside titanium alloy castings [5], as well as analysing stress distributions in girdled lizard osteoderms when a load is applied to simulate a bite of a predator [6].

Advanced morphological analysis was performed using the metrology toolbox of VGStudioMax 3.0. An advanced surface determination is used to find the material edge, after which various tools are used for different morphological analyses. In particular, the fang length was measured using a polyline with at least 10 points selected along the top of the fang from base to tip. As fang size variations occur also within species, the original skull belonging to each fang was also scanned using microCT, and skull length was measured from front to back as a relative size correction factor. In this way, relative fang size could be calculated from fang length/skull length. The polyline used to measure fang length was also used to fit a “best-fit” circle to the curvature of the fang, and the curvature was measured as the segment angle, i.e., the total angle covered by the fang on its best-fit circle. As the fang is a structure of varying thickness, a diameter value is difficult to calculate. In this work, the fang diameter was measured using a best-fit circle to the approximate middle of the fang in the cross-sectional slice image. A central section was selected by using a 10% region of interest around this mid-point of each fang and analysing that section for material fraction (BV/TV) and wall thickness analysis.

Physical compression tests were performed with a Deben CT500 microtest stage (500N max). The fang was glued to a polymer disk and placed on the top jaw of the stage, while a polymer disk was placed on the bottom jaw, with rigid foam on top of it. The fang was slowly moved toward the foam in compression mode at 0.2 mm/min, and the foam was pierced with no measured load (sensitivity ∼0.1 N). Live x-ray images were recorded of the compression process, and successful load tests were recorded for 2 fangs. Live x-ray videos are attached as Supplementary Material. For calculation of stress, the cross-sectional area of the fang at the failure location was taken. For calculation of strain, the total fang length was taken.

## Results and Discussion

**Figure 1:**
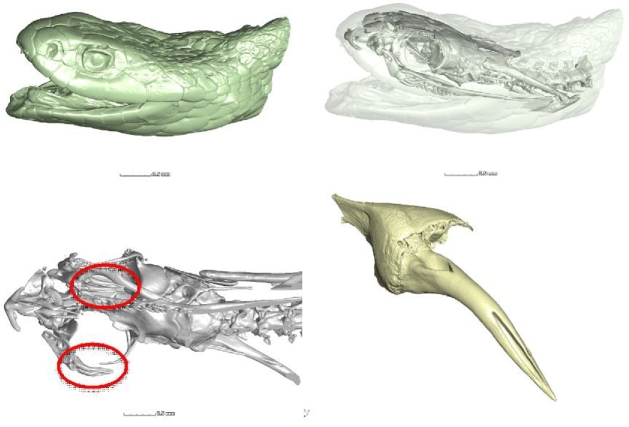
Location of fangs in night adder (*Causus rhombeatus*).

MicroCT images of the night adder *Causus rhombeatus* (NCBI Taxon ID: 44735) are shown in Fig. 1. The series of images, from left to right and top to bottom, show the whole-head microCT scan (first the exterior skin view, then a transparent view showing upper jawbone and skull, then rotated jawbone with circles indicating the location of fangs [including replacement fangs] in mobile anterior position). A high-resolution scan of 1 fang of this type is shown at the bottom right, with an entirely fused venom canal.

**Figure 2:**
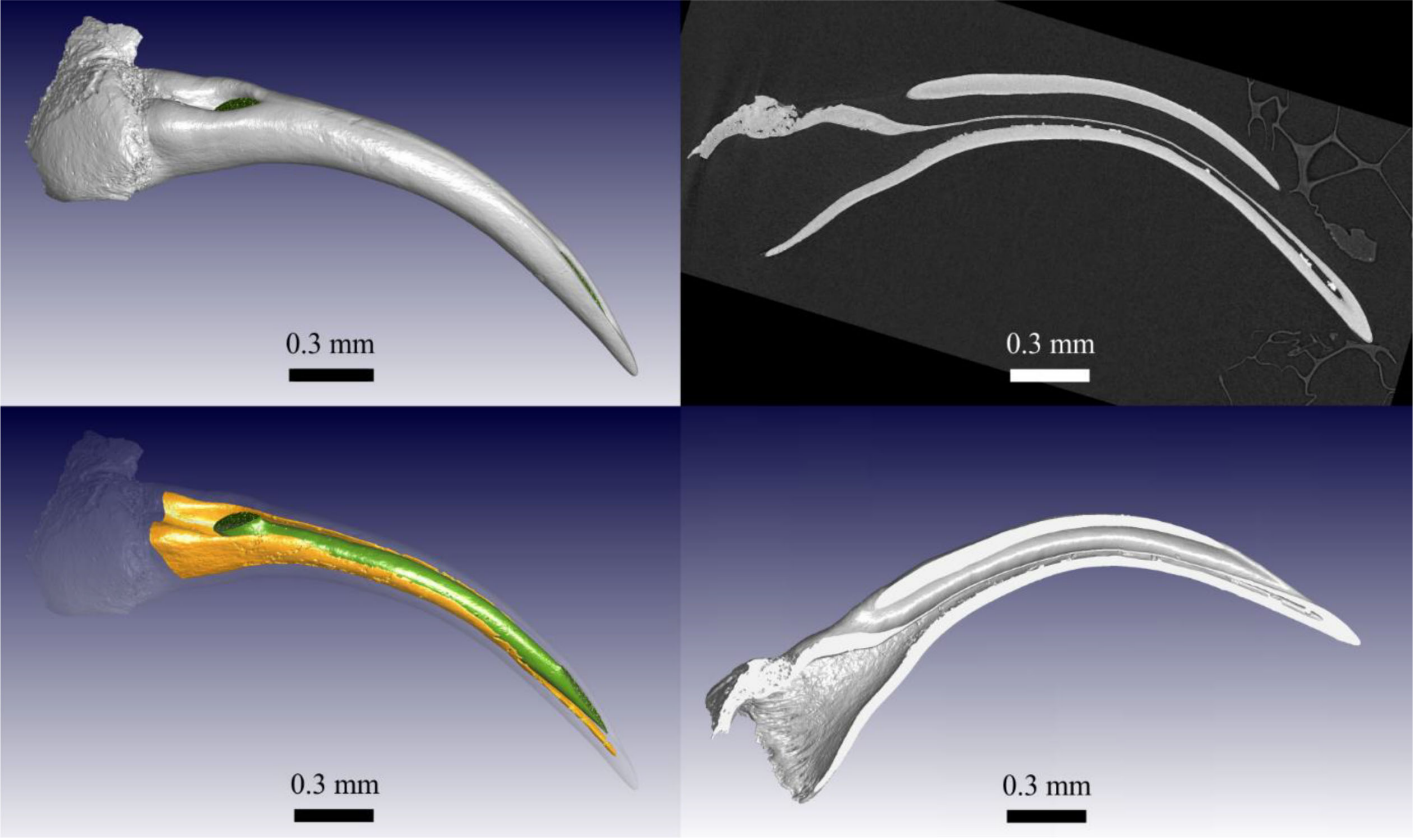
Internal structure of fang visualized using microCT data. Venom canal is in green and pulp cavity in orange in 3D view; sliced and cropped 3D views to the right show wall thickness and curvature of the structure.

A microCT scan of a fang allows viewing of internal structures such as the venom canal and the pulp cavity, as seen in Fig. 2, while a microCT slice image shows more detail of the structure (e.g., the thin wall between the venom canal and pulp cavity), and a cropped 3D view puts this into perspective. Considering that many fangs are very small (some <1 mm) and samples are rare, this nondestructive approach allows a unique insight into these types of structures, allowing slicing virtually at any angle.

**Figure 3:**
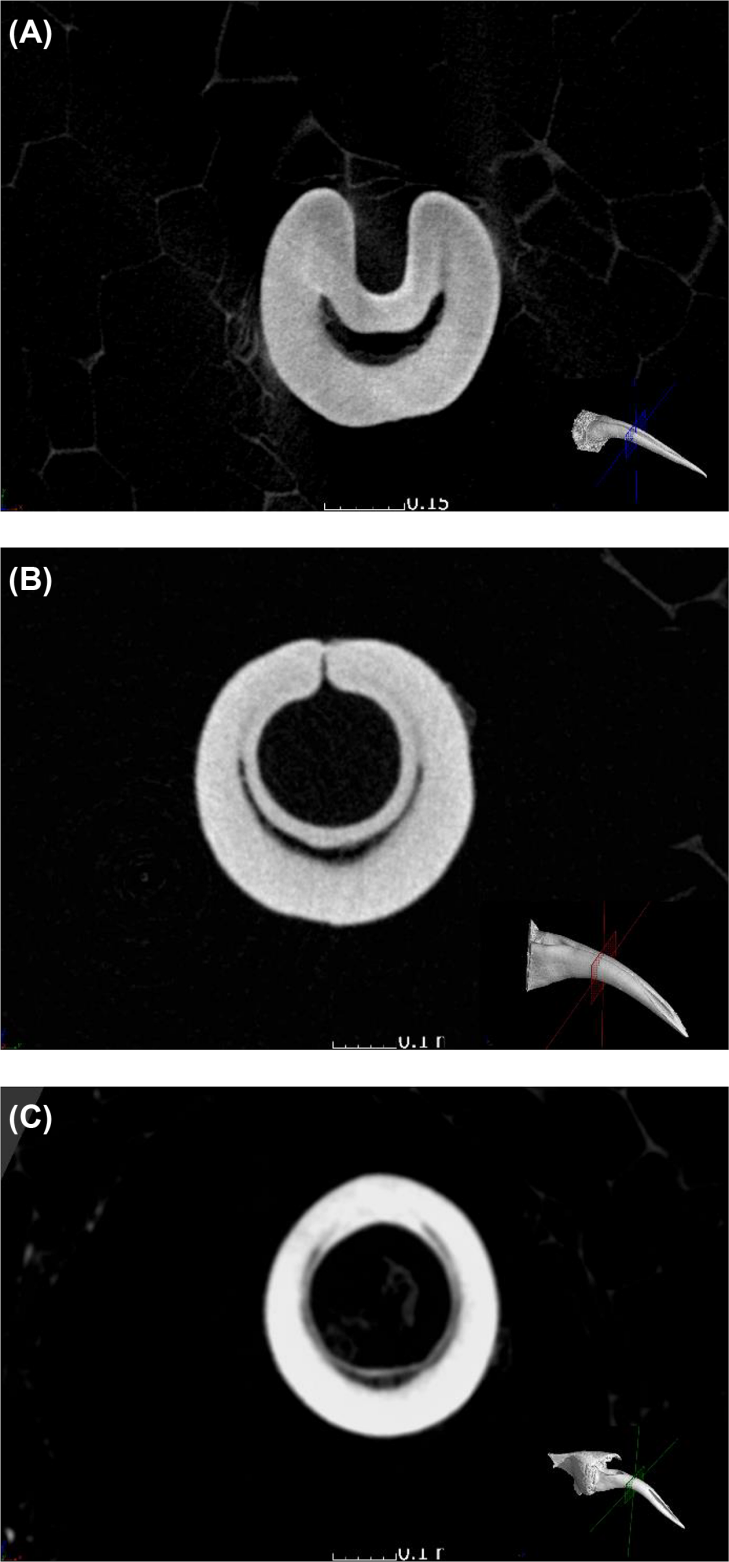
Cross-sectional slice images of 3 fang types: (**A**) open groove, (**B**) closed nonfused, and (**C**) closed fused phenotype.

The 3 types of fangs investigated are shown with representative examples in Fig. 3, with CT cross-sectional view also indicating the pulp cavity and venom canal.

**Figure 4:**
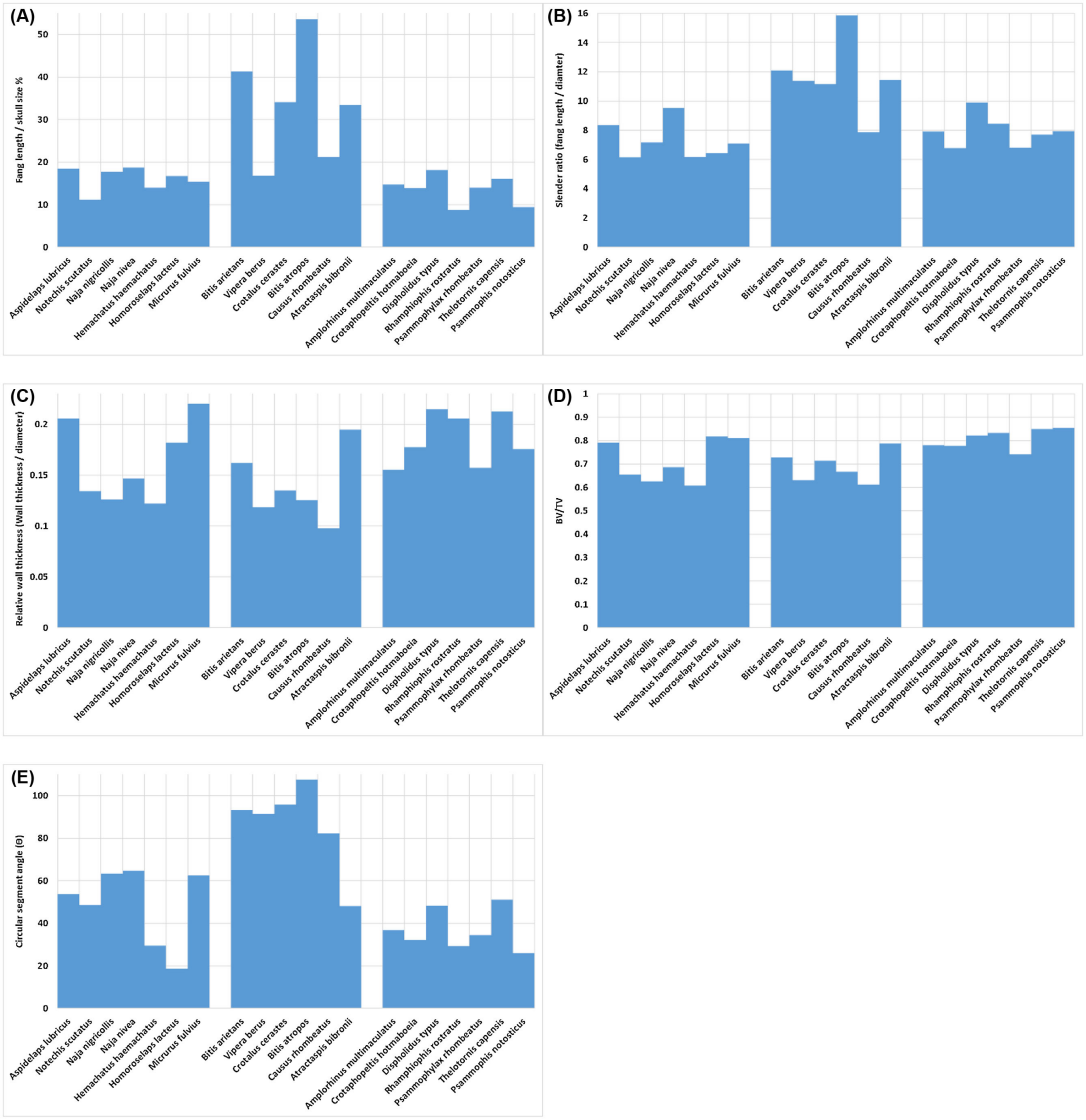
Morphological measurements obtained for 20 snake species (1 sample per species), grouped as (left) closed fangs with suture line, (middle) fused fang, and (right) open groove fangs. Morphometrics shown are (**A**) relative fang length, (**B**) fang length over diameter (slender ratio), (**C**) wall thickness over diameter (size-corrected wall thickness), (**D**) material volume fraction (BV/TV), (**E**) curvature measured as circular segment angle.

As many variations exist in fang morphology, a detailed analysis was conducted in an attempt to correlate morphological features with fang types. The first such measurement was the relative fang length. Because fang size depends highly on the size of the individual, skull length was calculated to correct for this. Each snake's skull was scanned, and its length was used to calculate a relative fang length. As seen in Fig. 4A, the closed fused fangs are slightly longer on average, while the open groove fangs are slightly shorter. The “slender ratio” is a measure of length in relation to fang total diameter, taken at the middle of the fang (Fig. 4B). In this case, again, the closed fused fangs seem more slender. The relative wall thickness (Fig. 4C) was calculated as the average wall thickness at the middle of the fang (10% of length of fang), in relation to the fang diameter at the middle (i.e., size corrected). The wall thickness is important as a thin wall will result in a weaker structure. However, the size-corrected wall thickness is very similar across all fang types, with open groove fangs having slightly thicker walls on average. A similar measure is the material volume fraction or BV/TV value (Fig. 4D), which is used widely in biomedical analysis, e.g., for trabecular bone. The middle section of the fang (the same as used for the wall thickness) was analysed for material fraction, including the venom canal, even in the open groove fang (using an advanced segmentation process). In this case, the volume fractions of material are similar, with the open groove fang type having a slightly higher material volume fraction. Finally, the curvature was measured using a method whereby the top curve of the fang was used to fit a circle, and the angle covered by the length of the fang on this circle was measured as the segment angle (Fig. 4E). A higher value indicates a higher curvature, with the closed fused fangs having the highest curvature and the open groove fangs having the lowest curvature on average.

**Figure 5:**
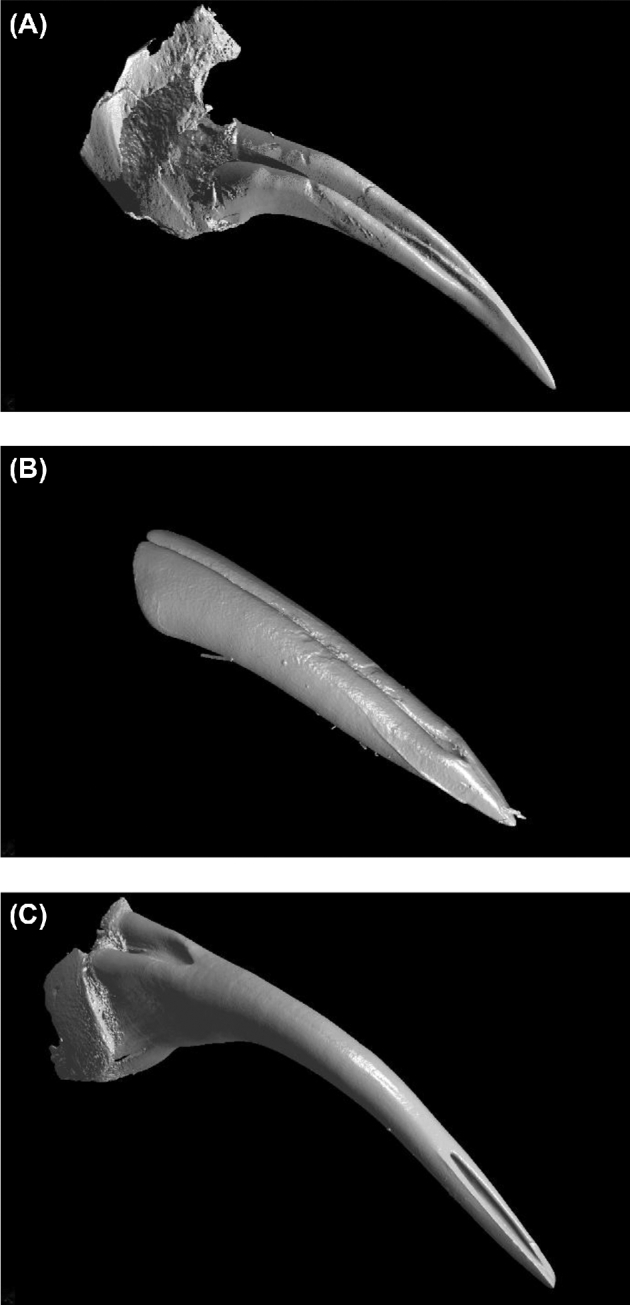
Sharp edges occurring in different places in different fang types shown here are representative examples of (**A**) long sharp edges along top and bottom of open groove fangs, (**B**) sharp edges around the horizontal sides of the tip of closed fangs, and (**C**) sharp edges along the top and bottom of the tip of entirely fused fangs.

All the above results indicate that closed fused fangs are relatively longer, more slender (i.e., long and thin), and more curved than other fang types. Open groove fangs are less curved and shorter but have thicker walls and higher material volume fractions, presumably to compensate for their smaller size. Large variations exist, as can be expected within each category. An interesting observation was that sharp edges are found on many fangs, most likely meant to assist in piercing. It was found that each fang type has a specific type of sharp edge associated with it. The open groove fangs have a long sharp ridge along the top and bottom of the fang running from the tip to more than half the fang length (Fig. 5A). The closed nonfused fangs have small ridges on each side of the tip laterally (Fig. 5B). The closed fused fangs have sharp edges only near the tip along the top and bottom, but extending only to the venom exit orifice (Fig. 5C). The larger edges found in the open groove fang type could be correlated to its posterior position in the maxilla and feeding behaviour that entails bite and hold (chew). This type of bite is expected to have a lower strike force, thereby requiring sharper and more pronounced edges to assist in breaking the skin of the prey. Both the open groove and closed fused types have sharp edges along the top and bottom, and both these types have mobile positions in the maxilla. The mobility allows a wider range of strike angles, and the vertical edges might be more effective over more angles. The closed unfused type is found in a fixed anterior position and has lateral edges. It can be imagined that once a bite has taken place and the fang is embedded in the prey, it may be subjected to lateral forces. Presumably, the lateral blades assist in removal of the fang in such situations.

**Figure 6:**
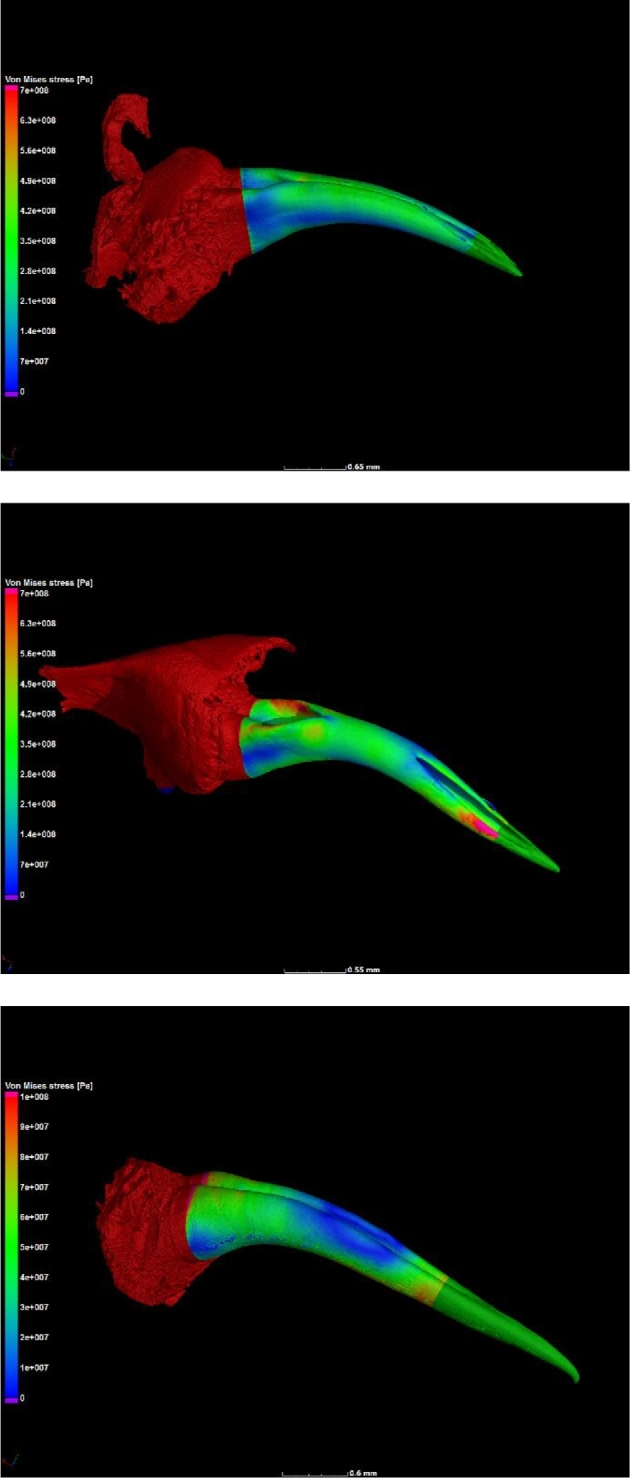
Von Mises stress distributions visualized for every fang type, with videos in the Supplementary Material. In order: *Naja nivea, Causus rhombeatus, Dispholidus typus*.

**Figure 7:**
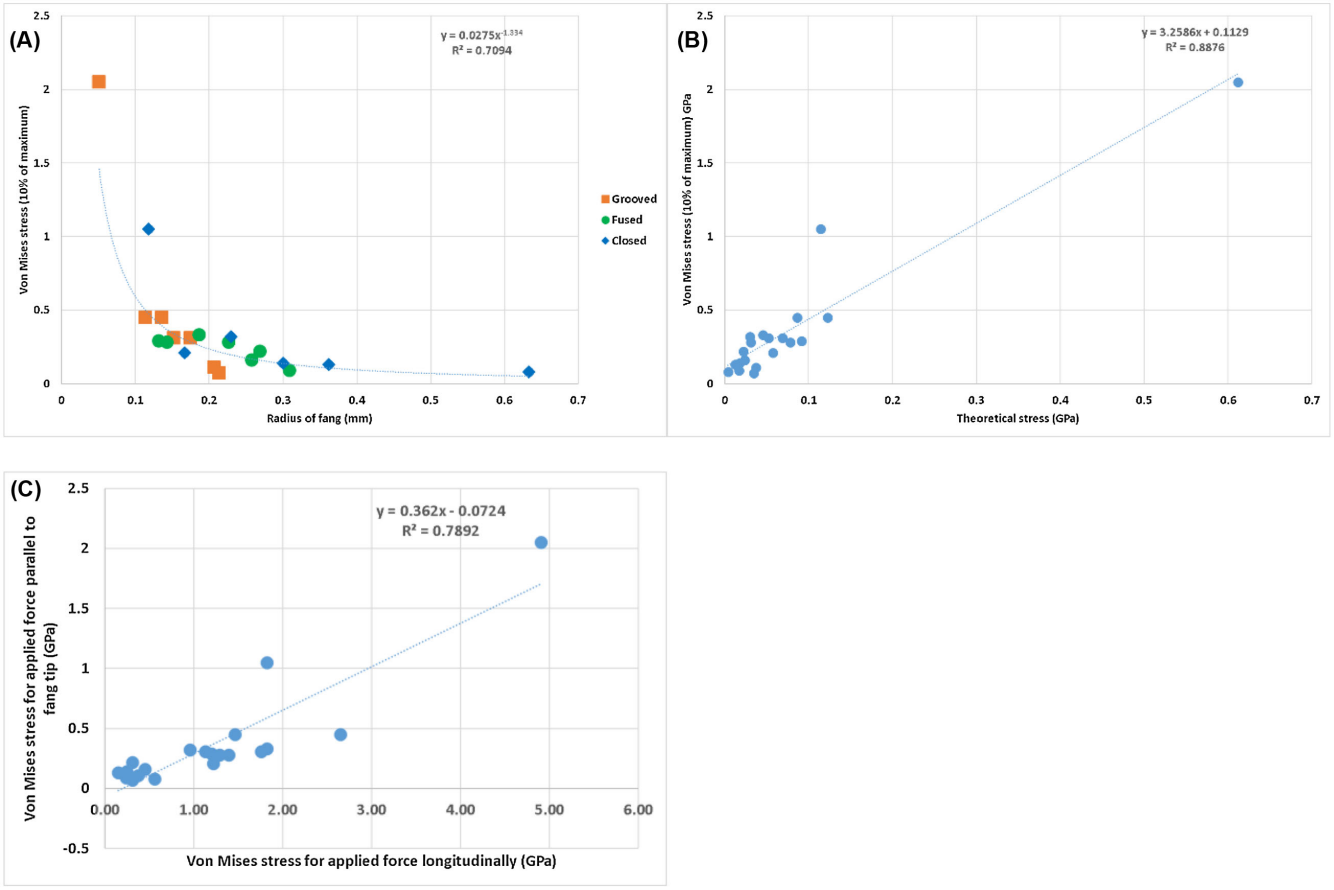
Von Mises stress values shown as a function of (**A**) fang middle radius [1] and (**B**) theoretically calculated stress for a rod of the same radius with measured material volume fraction. (**C**) Stress values for parallel load compared with those for lateral loads.

In order to directly compare the structural mechanics of the fang phenotypes, taking all morphological parameters directly into account, image-based load simulation was performed on each fang. A fixed load was applied to the tip of every fang with its base held in place. The resulting Von Mises stress was visualized as shown in Fig. 6 and measured in a 10% interval at maximum in the statistical results for each simulation. As fang sizes differ, the results are expected to depend on fang radius with a power law. This is shown in Fig. 7A for each fang type indicated, from data in Broeckhoven and du Plessis [1]. By using the fang diameter at the middle and calculating a theoretical stress value for the same force applied in the simulation, a simplified theoretical stress value could be calculated for each fang (corrected for differences in material volume fraction, neglecting the curvature and the cone shape). By showing the simulation stress results in comparison with theoretical stress values (Fig. 7B), it can be shown that all fangs have shapes that respond similarly to applied static loads and no fang types are unexpectedly stronger or weaker than others due to their shape or internal cavity sizes, wall thickness, or combinations of morphological factors. In addition, simulations were performed with the load applied laterally to the tip (at 90 degrees), and the maximum stresses were recorded. These maximum stresses correlate linearly with maximum stress for the parallel load as shown in Fig. 7C, indicating that all fangs are equally strong laterally (and none are weaker than others for lateral loads). The lateral loads cause an increase in stress by a factor of 3 compared with linear loading.

**Figure 8:**
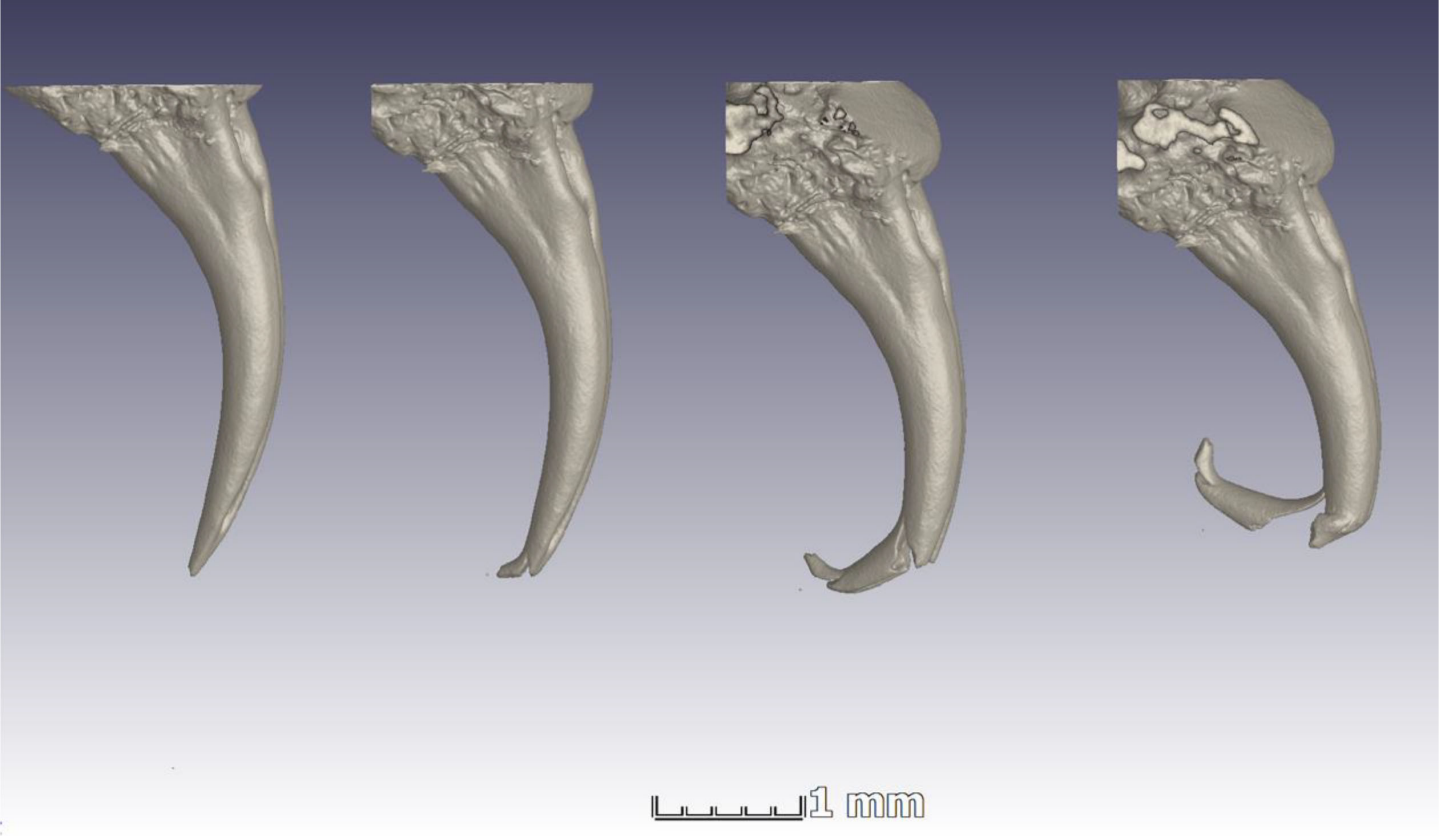
A sequence of microCT scans showing progressive failure in a single *Naja nivea* fang.

In an effort to validate the simulation results, dried, nonpreserved fangs were subjected to mechanical load tests. In Fig. 8, a sequence of microCT images shows sequential loading and imaging, showing the failure occurring first at the tip and then near the top of the venom canal exit orifice.

**Figure 9:**
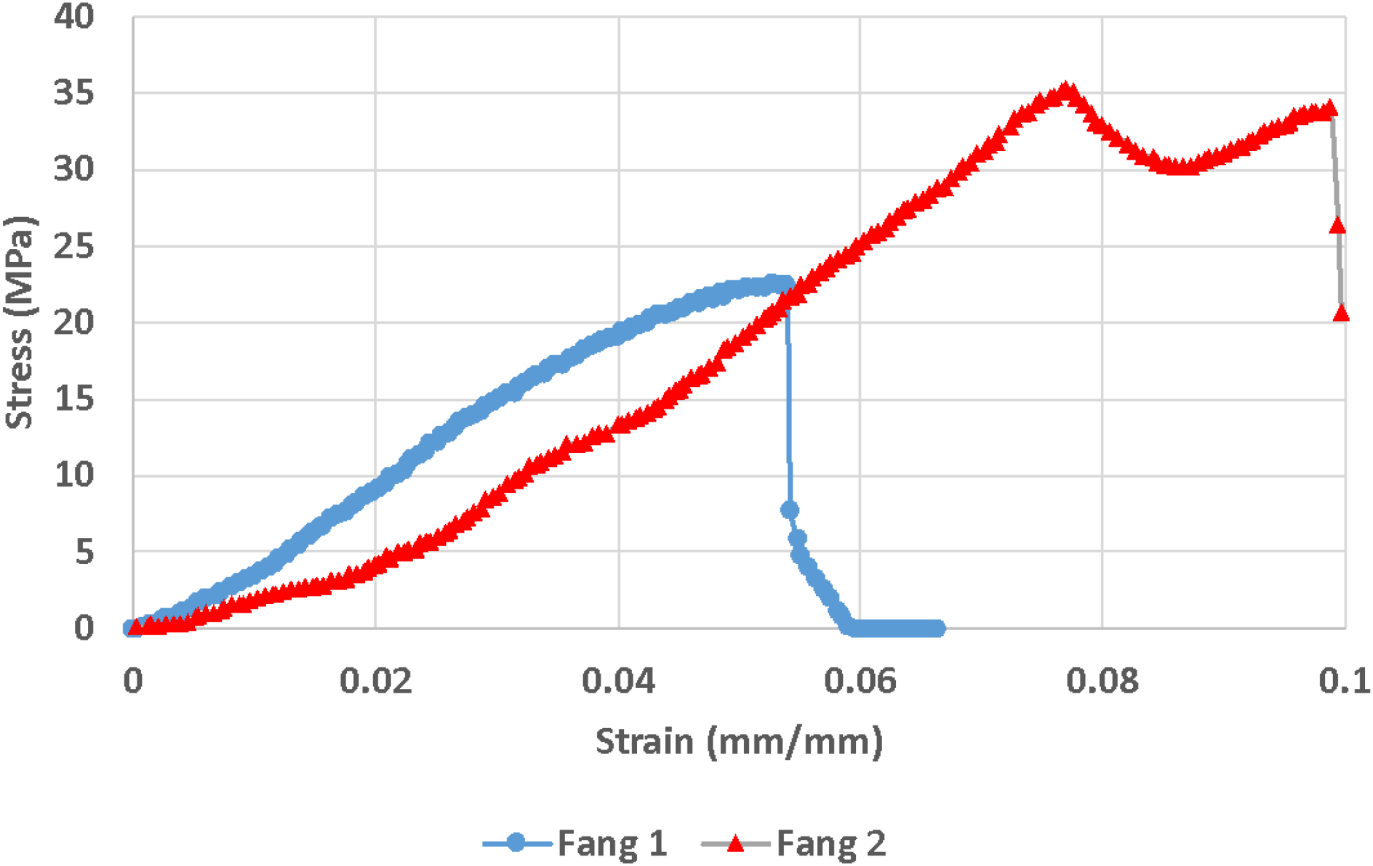
Stress strain curves obtained for 2 fangs of *Naja nivea*, different specimens than the 1 shown in Fig. 8. Both of these curves were obtained during live x-ray imaging, with both videos available in the Supplementary Material.

Mechanical loading to failure was successfully completed for 2 fangs. It was found that the maximum force at yield is between 2 and 4 N. This is surprisingly low even considering the small size of the fangs (5 mm). Stress strain curves were obtained and are shown in Fig. 9, indicating that the yield stress is near 25–35 MPa and the Young's modulus (of the entire structure including cavities) is ∼500 MPa.

These values allow an estimation of the material Young's modulus using the material volume fraction and assuming the material acts as an open cell foam. Initial simulations using 20 GPa for Young's modulus of the fang material result in much higher estimation of the effective Young's modulus of the entire structure. A lower value of 1.25 GPa was thus estimated and applied in the simulation of this fang type. The resulting displacement found by simulation allows calculation of the effective Young's modulus of the entire structure, 365 MPa in this case. This value of Young's modulus is therefore more reasonable (corresponding roughly to the 500 MPa obtained by compression testing). This value of ∼1.25 GPa, which is the average Young's modulus of the fang material, is much lower than the 20 GPa found by indentation in previous studies. This highlights the possibility that the elastic modulus varies locally across the fang, and especially that it might be higher on the surface (where indentation normally takes place) or might vary between species as well.

## Conclusions

Venomous snake fangs were analysed by microCT using advanced morphological analysis and structural mechanics simulations. It was found that the 3 fang phenotypes that occur in various lineages of snakes all had distinctive characteristics besides the morphology of the venom-conducting canal. The open groove fangs appear to be shorter and less curved, while closed, fused fangs are longer, relatively thin, and more curved. Sharp edges are located in different places in each fang type, and could be correlated to bite behaviour. Incorporating all morphological information, structural mechanics simulations were performed on the microCT data. Results obtained in the form of stress values indicate that fang types all respond similarly to applied loads, both parallel and laterally. Lateral loads induce stresses 3 times higher than parallel loads. Physical compression tests were conducted on 2 snake fangs. Stress strain curves recorded for these 2 fangs allow calculation of elastic modulus of the fang structure (500 MPa) including its venom canal and pulp cavity. The location of failure in physical tests correlates well with the stress distributions from load simulations. These results indicate that the piercing and cutting ability of fangs is pivotal to their success, as the fangs do not appear to be physically very strong (yield stress ∼25–35 MPa).

## Availability of supporting data

Supporting microCT data are available as image stacks and STL files from the *GigaScience* database (*Giga*DB), alongside videos of snake fang physical compression testing [7].

## Abbreviations

3D: 3-dimensional; CT: computed tomography; Pa: pascal; ROI: regions of interest.

## Competing interests

A. du Plessis and S.G. le Roux manage and operate the Stellenbosch University CT Facility.

## Supplementary Material

GIGA-D-17-00198_Original_Submission.pdf

GIGA-D-17-00198_Revision_1.pdf

GIGA-D-17-00198_Revision_2.pdf

Response_to_Reviewer_Comments_Original_Submission.pdf

Response_to_Reviewer_Comments_Revision_1.pdf

Reviewer_1_Report_(Original_Submission) -- Chris Armit 31 Aug 2017 Reviewed

Reviewer_1_Report_(Revision_1) -- Chris Armit 22 Nov 2017 Reviewed

Reviewer_2_Report_(Original_Submission) -- Ed Stanley 17 Nov 2017 Reviewed

Supplemental material
