# Supplementary material for: Snake fangs: 3D morphological and mechanical analysis by microCT, simulation, and physical compression testing
Source: Gigascience. 2017 Dec 15;7(1):gix126. doi: 10.1093/gigascience/gix126 (PMC5765556; doi:10.1093/gigascience/gix126)

GigaScience

Snake fangs: 3D morphological and mechanical analysis by microCT, simulation and physical compression testing

--Manuscript Draft--

**Manuscript Number:**	GIGA-D-17-00198
**Full Title:**	Snake fangs: 3D morphological and mechanical analysis by microCT, simulation and physical compression testing
**Article Type:**	Data Note
**Funding Information:**	
**Abstract:**	This data note provides data from an experimental campaign to analyse the detailed internal and external morphology and mechanical properties of venomous snake fangs. The aim of the experimental campaign was to investigate the evolutionary development of three fang phenotypes and investigate their mechanical behaviour. The study involved the use of load simulations to compare maximum Von Mises stress values, when a load is applied to the tip of the fang. The conclusions of this study has been published elsewhere, but in this data note we extend the analysis, providing morphological comparisons including details such as curvature comparisons, thickness, etc. Physical compression results of individual fangs were also used to calculate the effective elastic modulus of the entire snake fang for the first time. This elastic modulus is significantly lower than that expected from indentation experiments, highlighting the possibility that the elastic modulus is higher on the surface than in the rest of the material. The microCT data is presented in the form of STL files, which simplifies the handling of the data and allow its re-use for future morphological studies. These fangs might also serve as bio-inspiration for future hypodermic needles.
**Corresponding Author:**	Anton du Plessis<br><br>SOUTH AFRICA
**Corresponding Author Secondary Information:**	
**Corresponding Author's Institution:**	
**Corresponding Author's Secondary Institution:**	
**First Author:**	Anton du Plessis
**First Author Secondary Information:**	
**Order of Authors:**	Anton du Plessis
	Chris Broeckhoven
	Stephan G le Roux
**Order of Authors Secondary Information:**	
**Opposed Reviewers:**	
**Additional Information:**	
**Question**	**Response**
Are you submitting this manuscript to a special series or article collection?	No
**Experimental design and statistics**<br><br>Full details of the experimental design and statistical methods used should be given in the Methods section, as detailed in our Minimum Standards Reporting Checklist.	Yes

Powered by Editorial Manager® and ProduXion Manager® from Aries Systems Corporation

Information essential to interpreting the data presented should be made available in the figure legends.<br><br>Have you included all the information requested in your manuscript?	
**Resources**<br><br>A description of all resources used, including antibodies, cell lines, animals and software tools, with enough information to allow them to be uniquely identified, should be included in the Methods section. Authors are strongly encouraged to cite Research Resource Identifiers (RRIDs) for antibodies, model organisms and tools, where possible.<br><br>Have you included the information requested as detailed in our Minimum Standards Reporting Checklist?	Yes
**Availability of data and materials**<br><br>All datasets and code on which the conclusions of the paper rely must be either included in your submission or deposited in publicly available repositories (where available and ethically appropriate), referencing such data using a unique identifier in the references and in the “Availability of Data and Materials” section of your manuscript.<br><br>Have you have met the above requirement as detailed in our Minimum Standards Reporting Checklist?	Yes

Manuscript

Click here to download Manuscript Fangs Gigascience Submit.docx

Data note:

Snake fangs: 3D morphological and mechanical analysis by microCT, simulation and physical compression testing

Anton du Plessis [1], Chris Broeckhoven [2], Stephan G. le Roux [1]

[1] CT Scanner Facility, Stellenbosch University, Stellenbosch, South Africa, 7602

[2] Dept of Botany and Zoology, Stellenbosch University, Stellenbosch, South Africa, 7602

Abstract

This data note provides data from an experimental campaign to analyse the detailed internal and external morphology and mechanical properties of venomous snake fangs. The aim of the experimental campaign was to investigate the evolutionary development of three fang phenotypes and investigate their mechanical behaviour. The study involved the use of load simulations to compare maximum Von Mises stress values, when a load is applied to the tip of the fang. The conclusions of this study has been published elsewhere, but in this data note we extend the analysis, providing morphological comparisons including details such as curvature comparisons, thickness, etc. Physical compression results of individual fangs were also used to calculate the effective elastic modulus of the entire snake fang for the first time. This elastic modulus is significantly lower than that expected from indentation experiments, highlighting the possibility that the elastic modulus is higher on the surface than in the rest of the material. The microCT data is presented in the form of STL files, which simplifies the handling of the data and allow its re-use for future morphological studies. These fangs might also serve as bio-inspiration for future hypodermic needles.

Introduction

The fangs of venomous snakes are highly modified for piercing the skin and ejecting venom into prey, providing them with a significant evolutionary and ecological advantage. Snake fangs vary considerably in size and shape and this morphological variation can be attributed to differences in body size, diet and feeding behaviour. In

advanced snakes, three types of venom-conducting fangs can be found: (1) closed fangs with enclosed venom conducting canal and suture line on top surface where two sides seem to close up, (2) entirely fused fangs with enclosed venom-conducting canal, and (3) open-groove fangs with venom ejected along the groove surface due to high viscosity of the venom.

In a recent experimental microCT campaign, we conducted a phylogenetically-informed analysis of fang phenotypes [1]. By using static load simulations applied to the microCT data of each fang, we found that, despite differences in shape and size, stress distributions after applying a load were similar between the three fang phenotypes. The results of the study suggest that fangs might be biomechanically optimized. This work elaborates on the idea and makes use of advanced morphological comparisons, more detailed load simulations and physical compression tests to validate the simulation results. The fang models used for simulations and for morphological measurements are included in the form of STL files. These STL files are significantly smaller than full microCT data sets, and provide dimensionally accurate 3D models of the fangs. This simplified format hopefully allows a wider usage of the dataset by other researchers.

Materials and methods

High resolution X-ray CT scans were recorded at the Stellenbosch University CT facility [2], using optimized parameters for highest quality scanning using nanoCT [3]. Voxel sizes were between 1-8 µm depending on fang size. Each fang was individually loading in a rigid foam in a vertical orientation, with the foam attached to a glass rod. Scan settings included 60 kV and 240 µA with fast-scan option, resulting in approx. 1 hr per sample scan time. Data sets were processed in VGStudioMax 3.0 and static load simulations were performed using the *Structural Mechanics Simulation* module. This module makes use of voxel-based load simulation, similar to finite element modelling, but without need for meshing of surfaces. In this work a nominal load of 5 N was applied to the tip of the fang (in a region covering roughly half the distance to the venom canal exit orifice) and applied along the direction of the tip. Young's modulus values were taken from literature are 20 GPa [4] and Poisson's ratio 0.3. The fang was held at its base and resulting stress distributions could be analysed visually and quantitatively using in this case a 10% of maximum interval from the statistical stress results. This method has recently been applied in a study of tensile stresses around defects inside titanium alloy castings [5] as well as analysing stress distributions in girdled lizard osteoderms when a load is applied to simulate a bite of a predator [6].

Advanced morphological analysis was performed using the metrology toolbox of VGStudioMax 3.0. An advanced surface determination is used to find the material edge, after which various tools are used for different morphological analyses. In particular, the fang length was measured using a polyline with at least 10 points selected along the top of the fang from base to tip. Since fang size variations occur also within species, the original skull belonging to each fang was also scanned using microCT, and skull length was measured from front to back, as a relative size correction factor. In this way relative fang size could be calculated from fang length / skull length. The polyline used to measure fang length was also used to fit a “best-fit” circle to the curvature of the fang, and the curvature was measured as the segment angle, ie. the total angle covered by the fang on its best-fit circle. Since the fang is a structure of varying thickness, a diameter value is difficult to calculate. In this work, the fang diameter was measured using a best-fit circle to the approximate middle of the fang in the cross-sectional slice image. A central section was selected by using a 10% region of interest around this mid-point of each fang, and analysing that section for material fraction (BV/TV) and wall thickness analysis.

Physical compression tests were performed with a Deben CT500 microtest stage (500N max). The fang was glued to a polymer disk, placed on the top jaw of the stage, while a polymer disk was placed on the bottom jaw, with rigid foam on top of it. The fang was slowly moved towards the foam in compression mode at 0.2 mm/min, the foam was pierced with no measured load (sensitivity approx. 0.1 N). Live X-ray images were recorded of the compression process and successful load tests were recorded for two fangs. Live X-ray videos are attached as supplementary material. For calculation of stress, the cross sectional area of the fang at the failure location was taken. For calculation of strain, the total fang length was taken.

Results and discussion

The series of images in Figure 1 shows the night adder (*Causus rhombeatus),* with whole-head microCT scan (skin view followed by transparent view showing upper jawbone and skull, then rotated jawbone with circles indicating the location of fangs (including replacement fangs) in mobile anterior position. A high resolution scan of one fang of this type is shown at the bottom-right, with entirely fused venom canal.

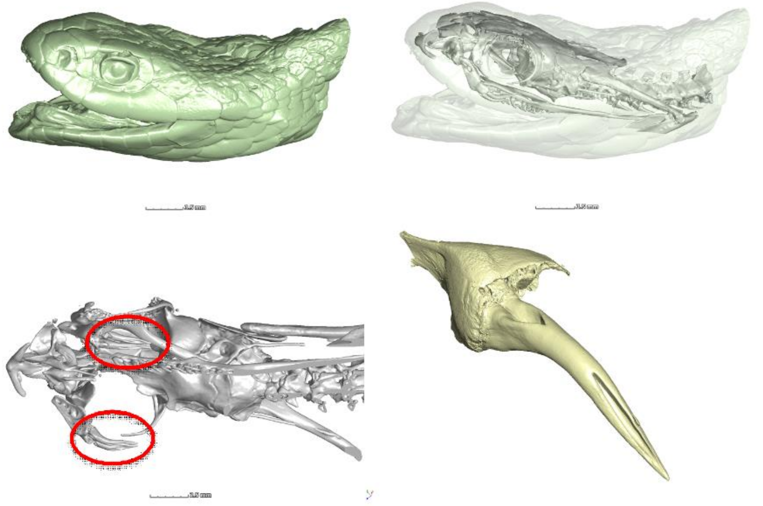

Figure 1: Location of fangs in night adder (*Causus rhombeatus*).

A microCT scan of a fang allows viewing of internal structures such as the venom canal and the pulp cavity as seen in Figure 2, while a microCT slice image shows more detail of the structure (eg. the thin wall between venom canal and pulp cavity) and a cropped 3D view puts this into perspective. Considering many fangs are very small (some < 1mm) and samples are rare, this non-destructive approach allows a unique insight into these types of structures, allowing slicing virtually at any angle.

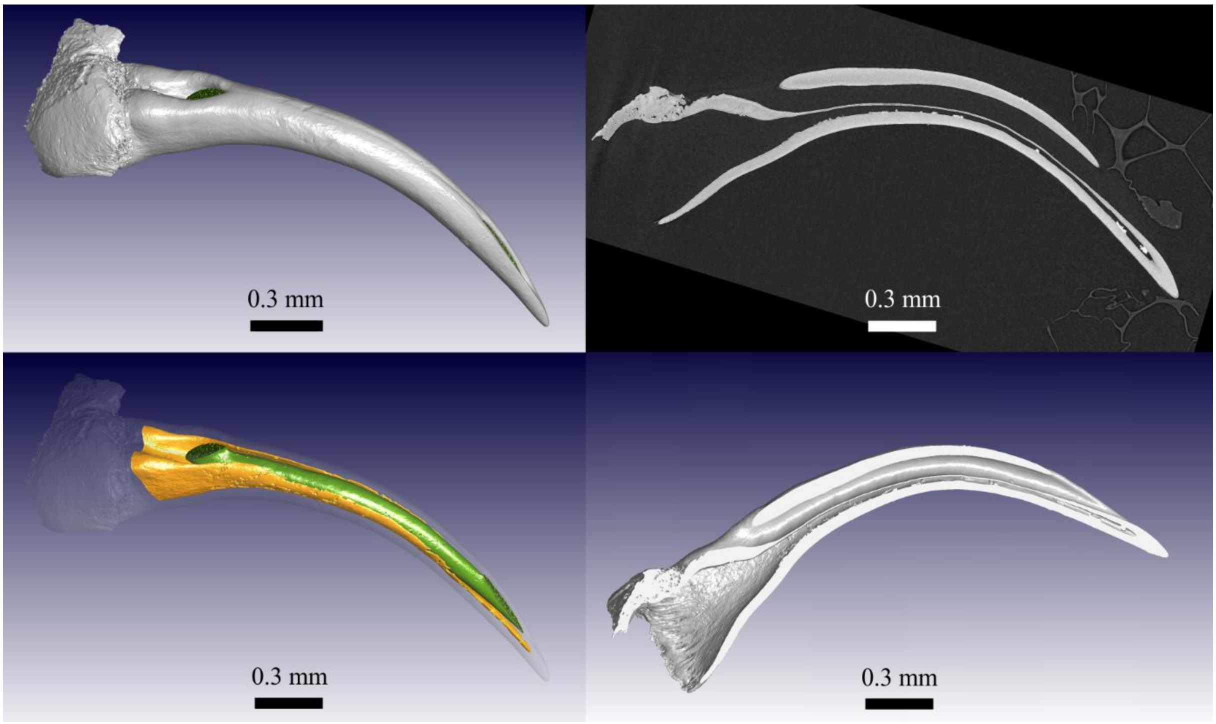

Figure 2: Internal structure of fang visualized using microCT data. Venom canal in green and pulp cavity in orange in 3D view, slice and cropped 3D views to the right show wall thickness and curvature of the structure.

The three types of fangs investigated are shown with representative examples in Figure 3, with CT cross sectional view also indicating the pulp cavity and venom canal.

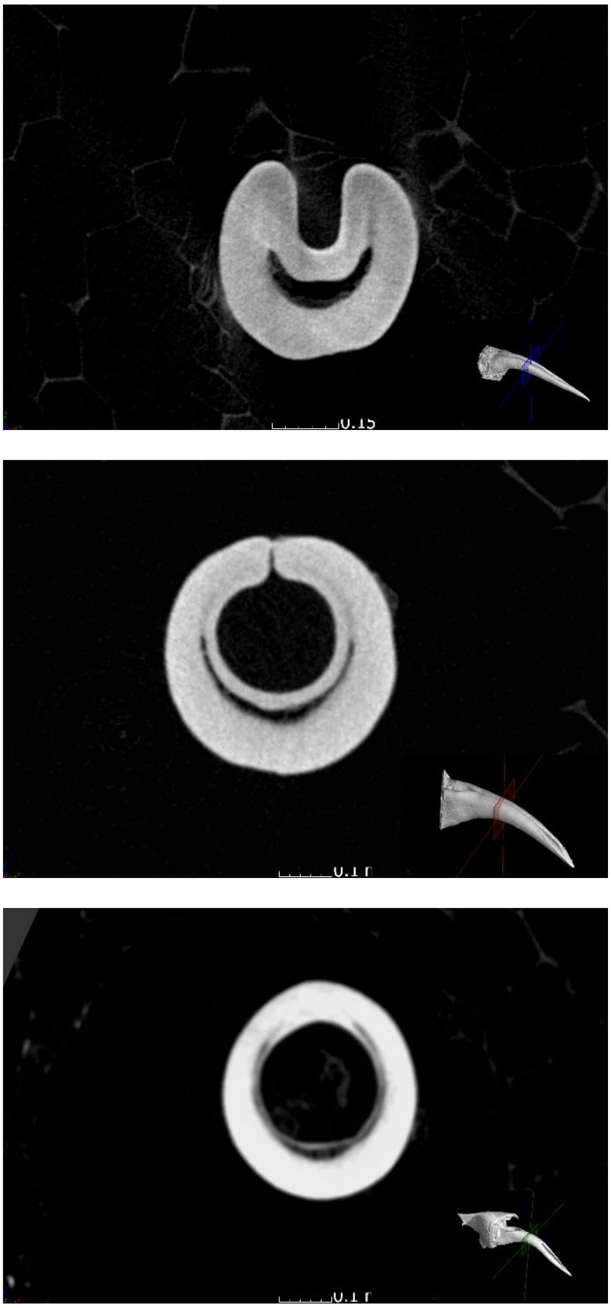

Figure 3: Cross-sectional slice images of three fang types: (a) open grooved, (b) closed non-fused and (c) closed fused phenotype.

Since many variations exist in fang morphology, detailed analysis was conducted in an attempt to correlate

morphological features with fang types. The first such measurement was the relative fang length. Since fangs size depends highly on the size of the individual, skull length was calculated to correct for this. Each snake's skull was scanned and its length used to calculate a relative fang length. As seen in Figure 4(a), the closed fused fangs have slightly longer fangs on average, while the open grooved fangs have slightly shorter fangs. The "slender ratio" is a measure of length in relation to fang total diameter, taken at the middle of the fang (Figure 4(b)). In this case again the closed fused fangs seem more slender. The relative wall thickness (Figure 4(c)) was calculated as the average wall thickness at the middle of the fang (10 % of length of fang), in relation to the fang diameter at the middle (ie. size corrected). The wall thickness is important as a thin wall will result in a weaker structure. However, the size-corrected wall thickness is very similar across all fang types, with open groove fangs having slightly thicker walls on average. A similar measure is the material volume fraction or BV/TV value (Figure 4(d)), which is used widely in biomedical analysis e.g. for trabecular bone. The middle section of the fang (the same as used for the wall thickness) was analysed for material fraction, including the venom canal even in the open groove fang (using an advanced segmentation process). In this case the volume fractions of material are similar, with the open groove fang type having a slightly higher material volume fraction. Finally, the curvature was measured using a method whereby the top curve of the fang was used to fit a circle, and the angle covered by the length of the fang on this circle was measured as the segment angle (Figure 4(e)). A higher value indicates a higher curvature, with the closed fused fangs having the highest curvature and the open-grooved fangs having the lowest curvature on average.

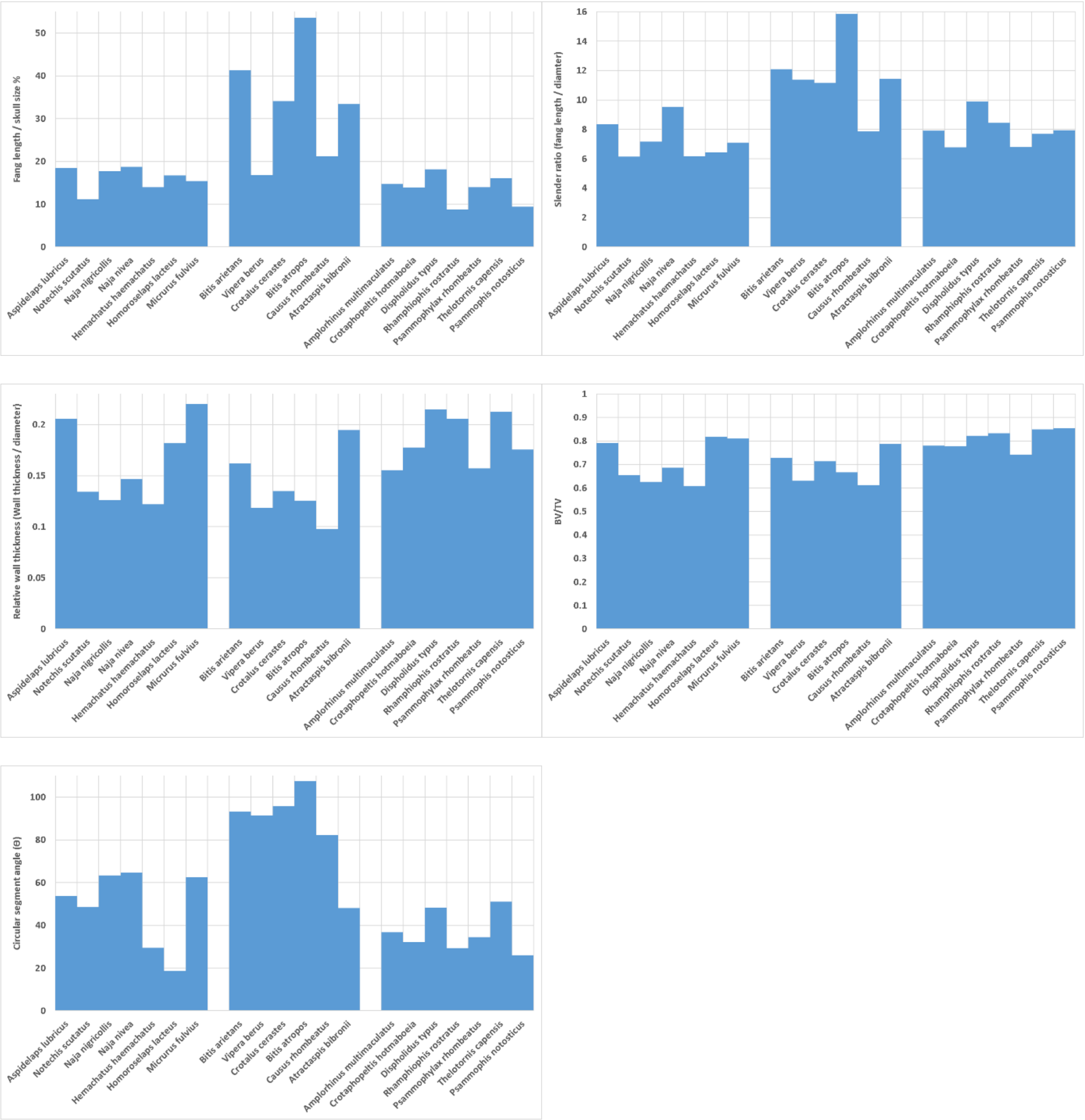

Figure 4: Morphological measurements obtained for 20 snake species, grouped as (left) closed fangs with suture line, (middle) fused fang and (right) open grooved fangs. Morphometrics shown are (a) relative fang length, (b) fang length over diameter (slender ratio), (c) wall thickness over diameter (size corrected wall thickness), (d) material volume fraction (BV/TV), (e) curvature measured as circular segment angle.

All the above results indicate that closed fused fangs are relatively longer, slender (i.e., long and thin) and more curved than other fang types. Open grooved fangs are less curved and shorter, but have thicker walls and higher material volume fractions, presumably to compensate for their smaller size. Large variations exist, as can be expected within each category. An interesting observation was that sharp edges are found on many fangs, most likely meant to assist in piercing. It was found that each fang type has a specific type of sharp edge associated with it. The open-grooved fangs have a long sharp ridge along the top and bottom of the fang running from tip to

more than half the fang length (Figure 5a). The closed non-fused fangs have small ridges on each side of the tip laterally (Figure 5b). The closed fused fangs have sharp edges only near the tip along the top and bottom but extending only to the venom exit orifice (Figure 5c). The larger edges found in the open-grooved fang type could be correlated to its posterior position in the maxilla, and feeding behaviour which entails bite and hold (chew). This type of bite is expected to be with a lower strike force, thereby requiring sharper and more pronounced edges to assist in breaking the skin of the prey. Both the open-groove and closed fused types have sharp edges along top and bottom, and both these types have mobile positions in the maxilla. The mobility allows a wider range of strike angles, and the vertical edges might be more effective over more angles. The closed unfused type with is found in a fixed anterior position has lateral edges. It can be imaged that once a bite has taken place and the fang is embedded in the prey, it may be subjected to lateral forces. Presumably the lateral blades assist in removal of the fang in such situations.

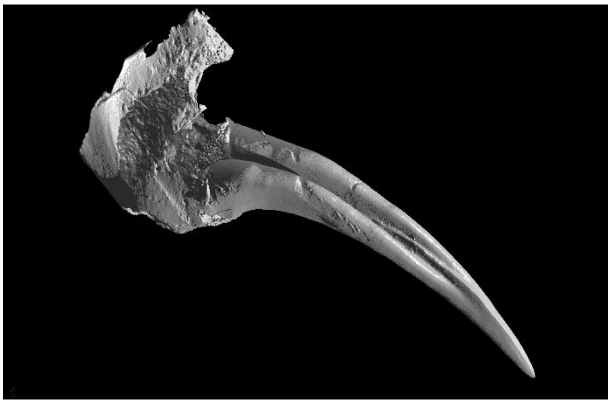

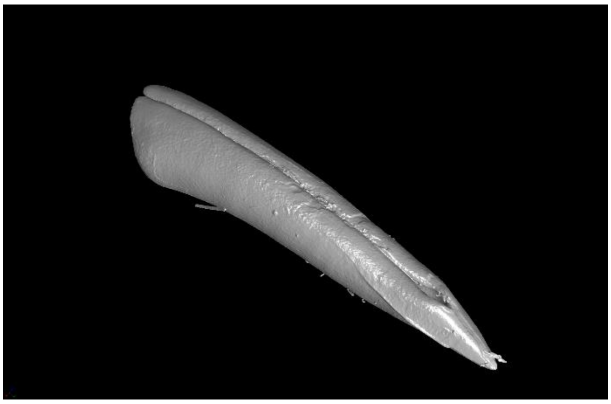

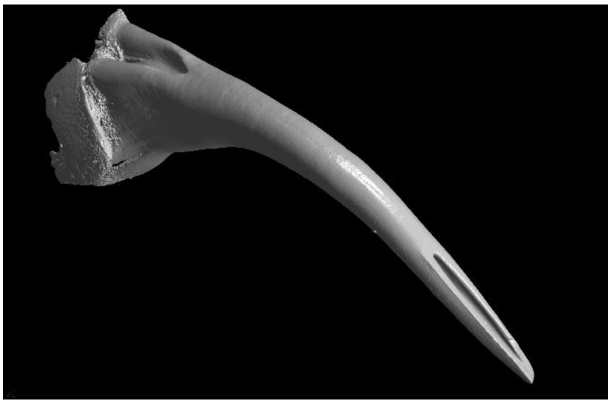

Figure 5: Sharp edges occurring in different places in different fang types, shown here are representative examples of (a) long sharp edges along top and bottom of open-grooved fangs, (b) sharp edges around the horizontal sides of the tip of closed fangs and (c) sharp edges along top and bottom of tip of entirely fused fangs.

In order to directly compare structural mechanics of the fang phenotypes, taking all morphological parameters directly into account, image-based load simulation was performed on each fang. A fixed load was applied to the tip of every fang with its base held in place. The resulting Von Mises stress was visualized as shown in Figure 6 and measured in a 10% interval at maximum in the statistical results for each simulation. Since fang sizes differ, the results are expected to depend on fang radius with a power law. This is shown in Figure 7a for each fang type indicated. By using the fang diameter at the middle and calculating a theoretical stress value for the same force applied in the simulation, a theoretical stress value could be calculated for each fang (corrected for differences in material volume fraction). By showing the simulation stress results in comparison to theoretical stress values (Figure 7b), it can be shown that all fangs have shapes that respond similarly to applied static loads and no fang types are unexpectedly stronger or weaker than others due to their shape or internal cavity wall thickness, or combinations of morphological factors. In addition, simulations were performed with load applied laterally to the tip (at 90 degrees) and the maximum stresses recorded. These maximum stresses correlate linearly with maximum stress for parallel load as shown in Figure 7c, indicating all fangs are equally strong laterally (and none are weaker than others for lateral loads). The lateral loads cause an increase in stress by a factor of 3.

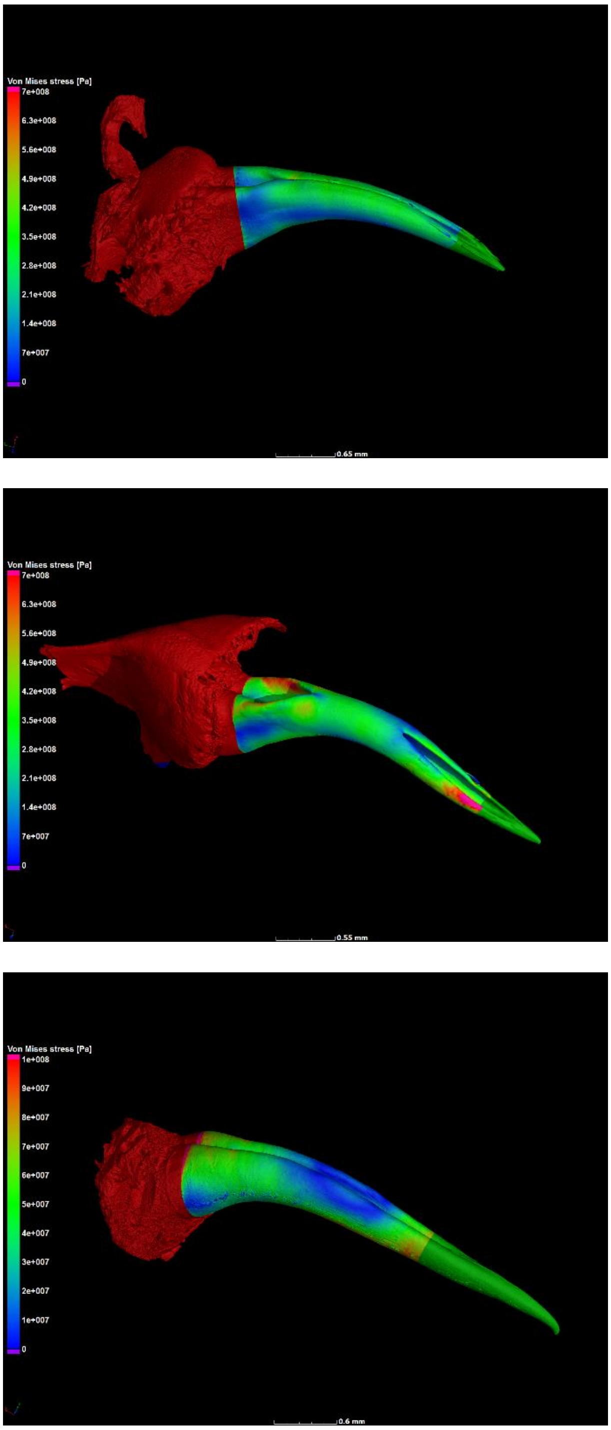

Figure 6: Von Mises stress distributions visualized for every fang type, with videos in supplementary material.

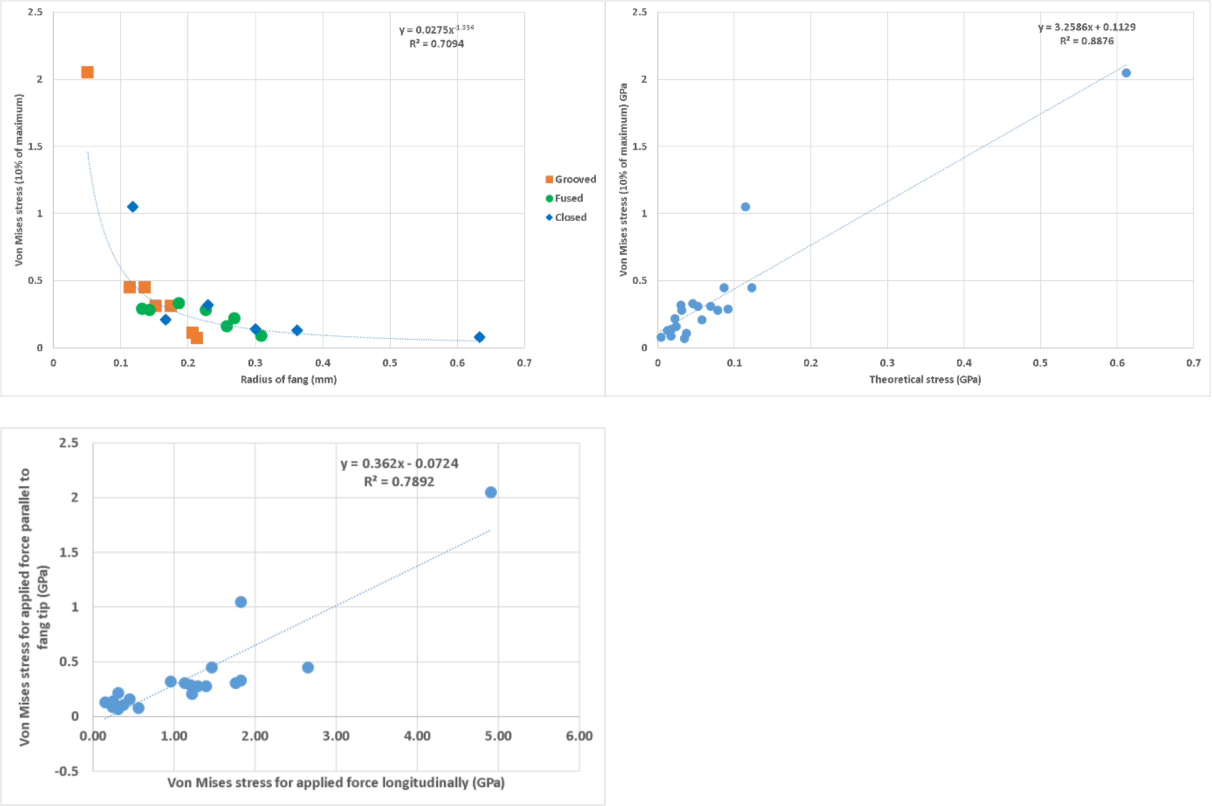

Figure 7: Von Mises stress values shown as a function of (a) fang middle radius, and (b) theoretically calculated stress for rod of same radius with measured material volume fraction, (c) shows stress values for parallel load compared to those for lateral loads.

In an effort to validate the simulation results, dried, non-preserved fangs were subjected to mechanical load tests. In Figure 8 a sequence of microCT images show sequential loading and imaging, showing the failure occurring first at the tip then near the top of the venom canal exit orifice.

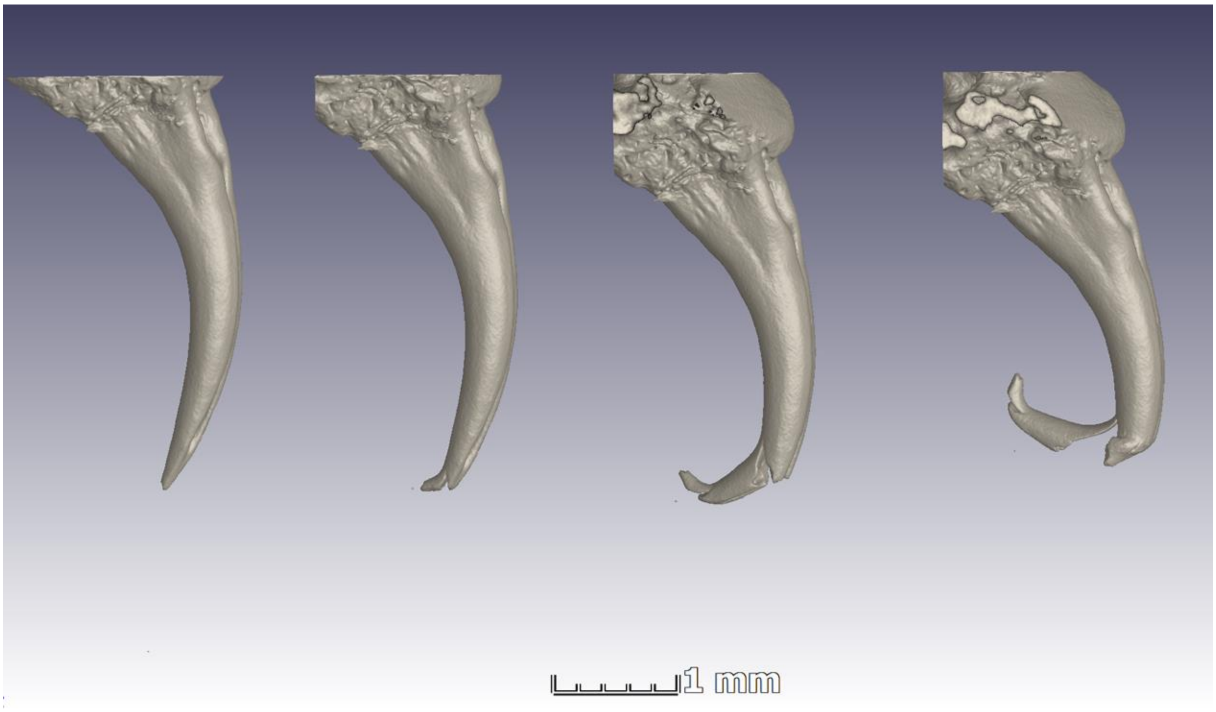

Figure 8: A sequence of microCT scans showing progressive failure

Mechanical loading to failure was successfully completed for two fangs. It was found that the maximum force at yield is between 2-4 N. This is surprisingly low even considering the small size of the fangs (5.2 mm). Stress-strain curves were obtained and one representative curve is shown in Figure 9, indicating the yield stress is near 25 MPa and the Young's modulus (of the entire structure including cavities) is 500 MPa.

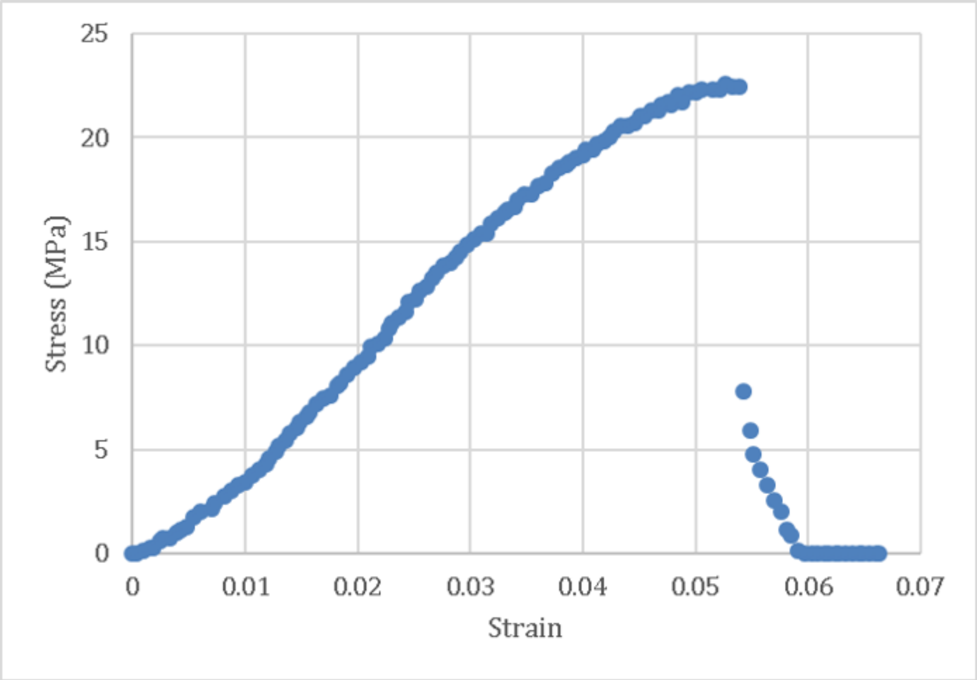

Figure 9: Stress-strain curve obtained for 5.2 mm fang

These values allow an estimation of the material Young's modulus, using the material volume fraction and assuming the material acts as an open-cell foam. A value of 1.25 GPa is estimated and was applied in the simulation of this fang type. The resulting displacement by simulation allows calculation of the effective Young's modulus of the structure, as 365 MPa in this case. This value of Young's modulus is therefore reasonable (corresponding to the 500N obtained by testing) and the simulations are validated by the experimental test, as an average for the entire fang. This value of ~ 1.25 GPa is much less than the 20 GPa found by indentation in previous studies, highlighting the possibility that the elastic modulus varies across the fang and especially is stiffer on the surface (where indentation normally take place).

Conclusions

Venomous snake fangs were analysed by microCT, using advanced morphological analysis and structural mechanics simulations. It was found that the three fang phenotypes which occur in various lineages of snakes all have distinctive characteristics besides the morphology of the venom-conducting canal. The open-grooved fangs appear to be shorter and less curved while closed, fused fangs are longer, relatively thin and more curved. Sharp edges are located in different places in each fang type, and could be correlated to bite behaviour. Incorporating all morphological information, structural mechanics simulations were performed on the microCT data. Results obtained in the form of stress values, indicate fang types all respond similarly to applied loads, both parallel and laterally. Lateral loads induce stresses 3 times higher than parallel loads. Physical compression tests were conducted for the first time on a snake fang. Stress-strain curves recorded for these two fangs allows calculation of elastic modulus of the fang structure (500 MPa) including its venom canal and pulp cavity. The location of failure in physical tests correlates well with the stress distributions from load simulations. These results indicate that the piercing and cutting ability of fangs is pivotal to their success, as the fangs do not appear to be physically very strong (Yield stress ~ 25 MPa).

Competing interests

The authors have no competing interests

References

1. Broeckhoven, C., & du Plessis, A. (2017). Has snake fang evolution lost its bite? New insights from a structural mechanics viewpoint. Biology Letters, in press.
2. du Plessis, A., le Roux, S. G., & Guelpa, A. (2016). The CT Scanner Facility at Stellenbosch University: an open access X-ray computed tomography laboratory. Nuclear Instruments and Methods in Physics Research Section B: Beam Interactions with Materials and Atoms, 384, 42-49.
3. Du Plessis, A., Broeckhoven, C., Guelpa, A., & Le Roux, S. G. (2017). Laboratory X-ray micro-computed tomography: a user guideline for biological samples. GigaScience, 6(6), 1-11.
4. Jansen van Vuuren, L., Kieser, J. A., Dickenson, M., Gordon, K. C., & Fraser- Miller, S. J. (2016). Chemical and mechanical properties of snake fangs. Journal of Raman Spectroscopy, 47(7), 787-795.
5. du Plessis, A., Yadroitsava, I., le Roux, S. G., Yadroitsev, I., Fieres, J., Reinhart, C., & Rossouw, P. (2017). Prediction of mechanical performance of Ti6Al4V cast alloy based on microCT-based load simulation. Journal of Alloys and Compounds.
6. Broeckhoven, C., du Plessis, A., & Hui, C. (2017). Functional trade-off between strength and thermal capacity of dermal armor: insights from girdled lizards. Journal of the Mechanical Behavior of Biomedical Materials.

Figure 1

Click here to download Figure Figure 1.tif

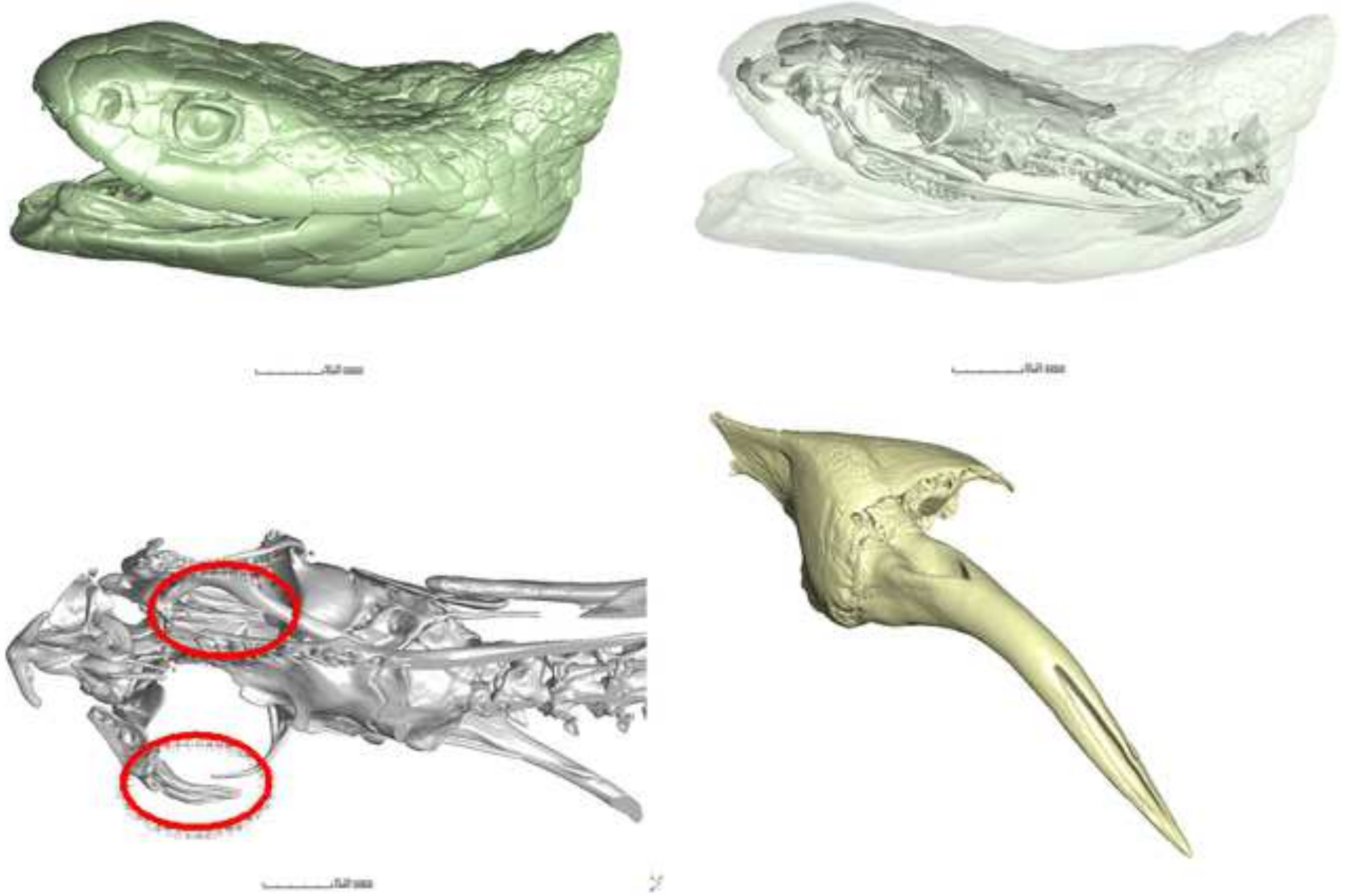

 Click here to download Figure Figure 2_new.tif

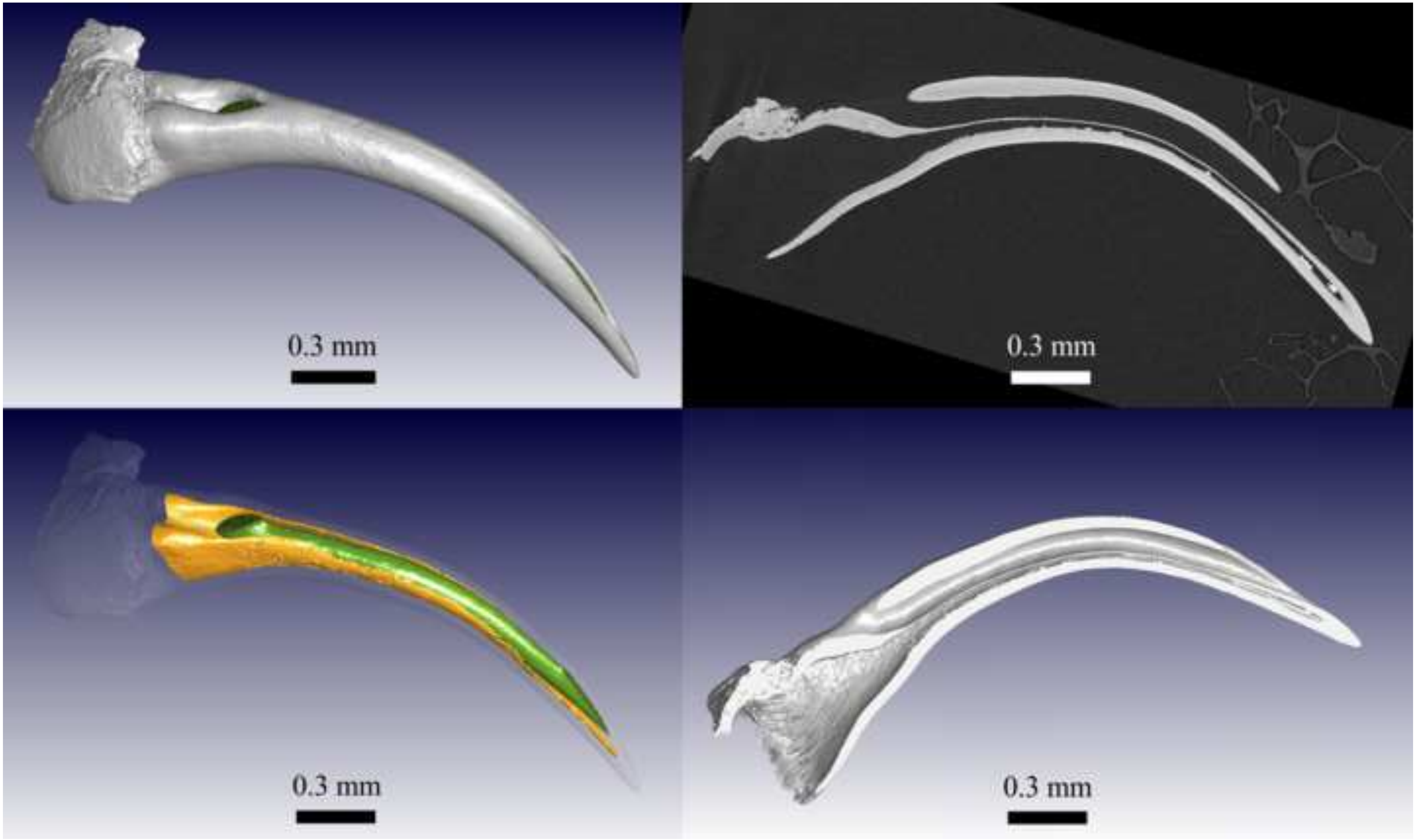

Figure 3A

Click here to download Figure Fig3A.tif

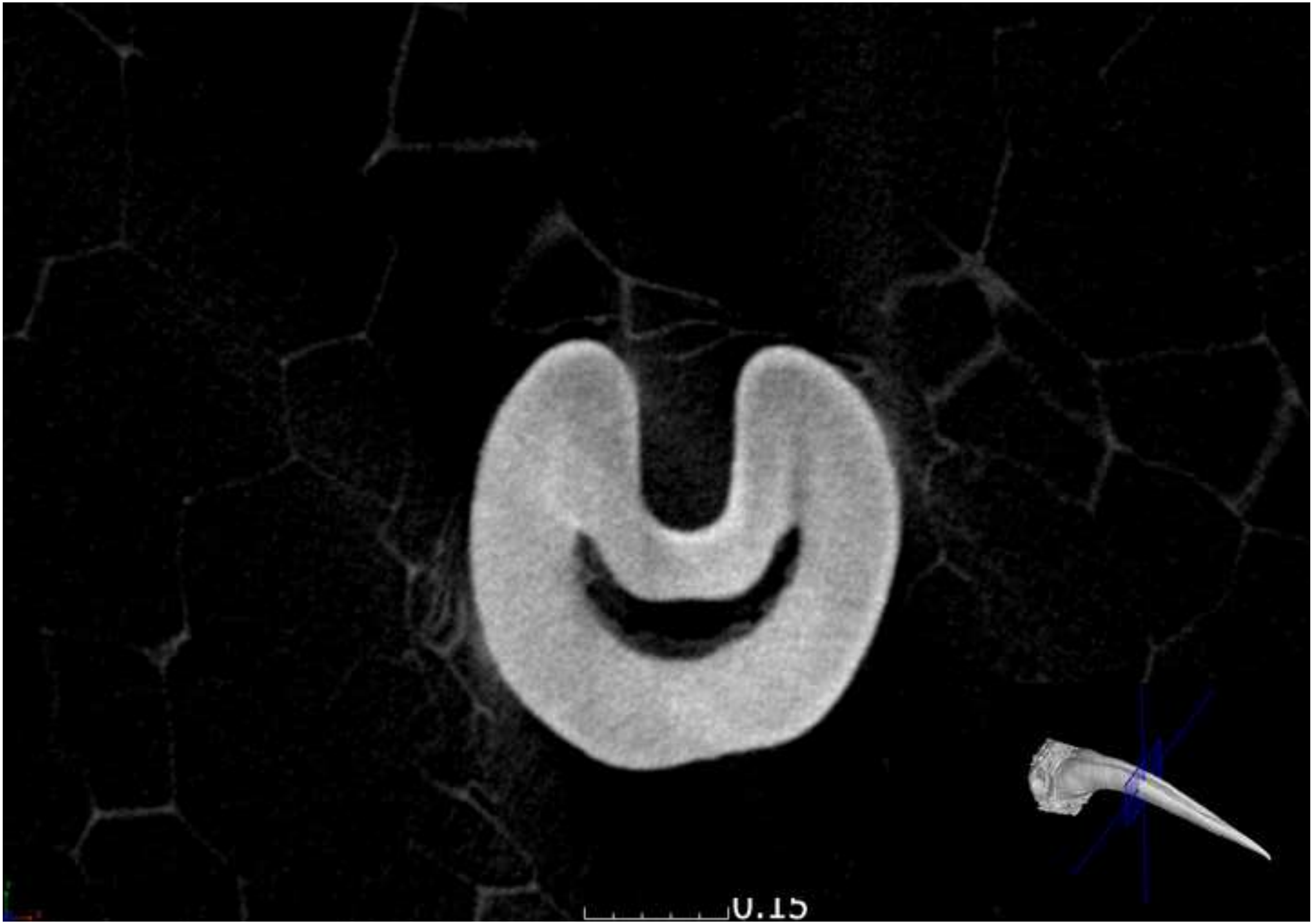

Figure 3B

Click here to download Figure Fig3B.tif

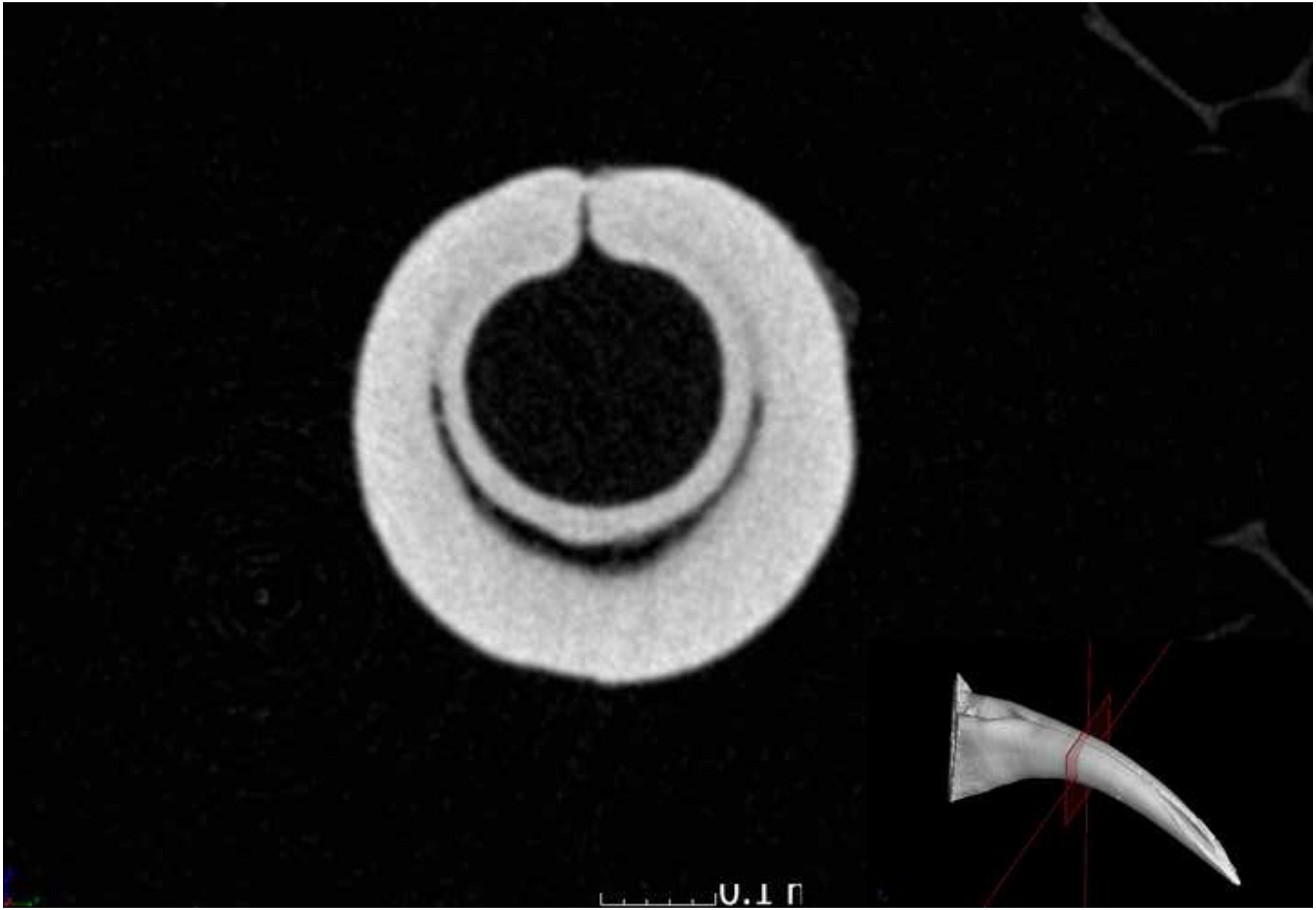

Figure 3C

Click here to download Figure FIg3C.tif

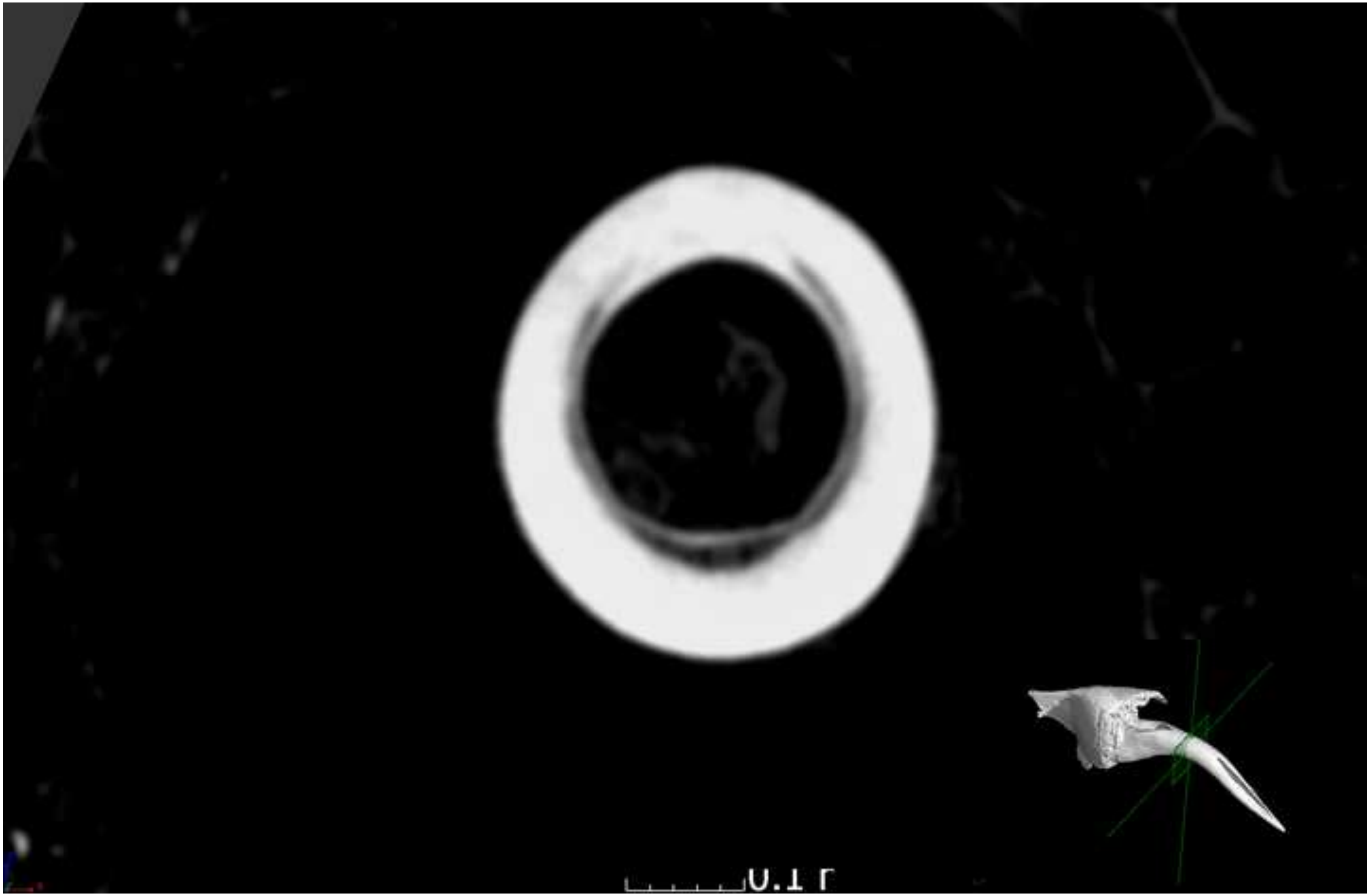

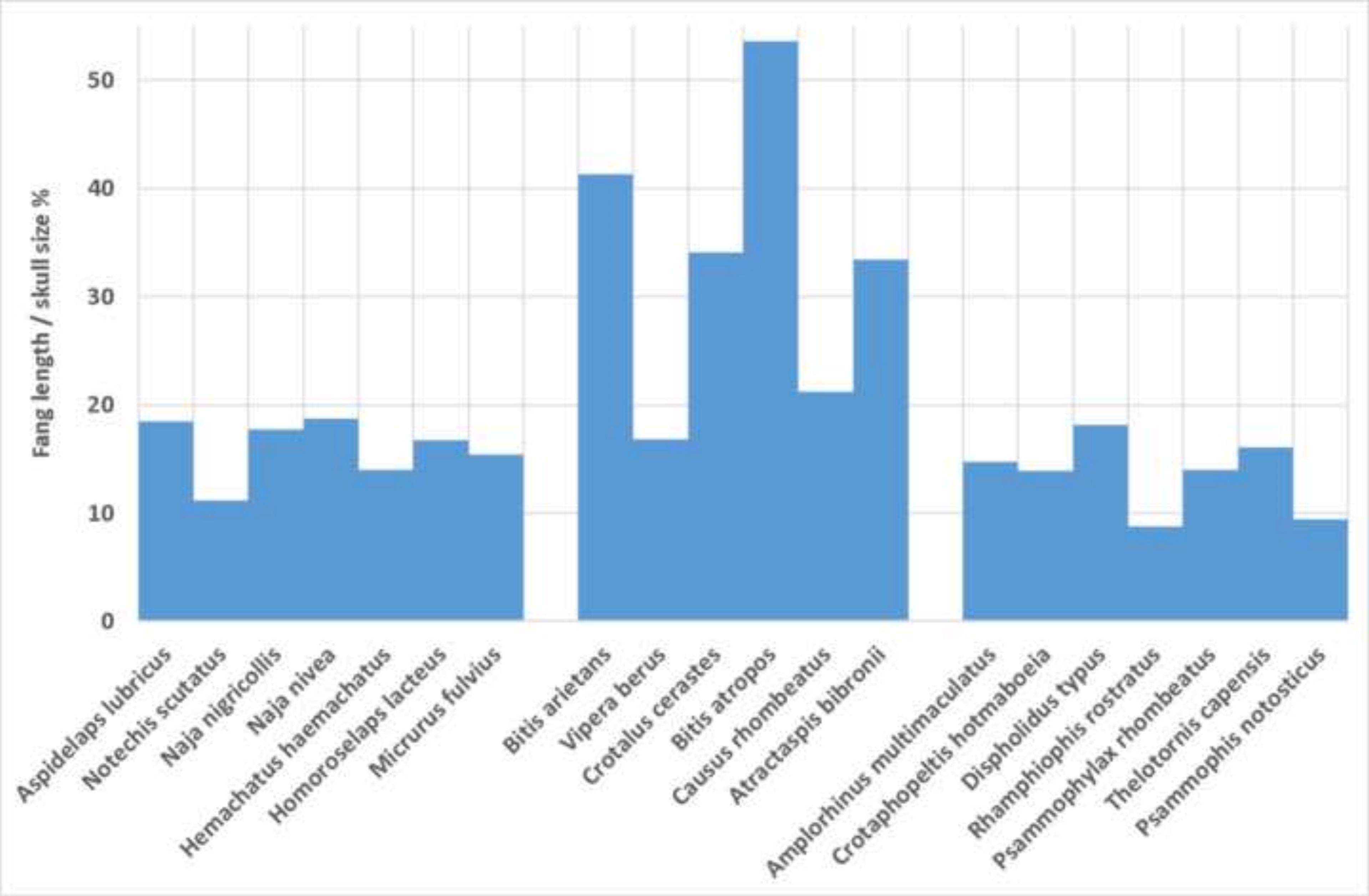
Fang length / skull size %
0
10
20
30
40
50
Aspidelaps lubricus
Notechis scutatus
Naja nigricollis
Naja nivea
Hemachatus haemachatus
Homoroselaps lacteus
Micrurus fulvius
Bitis arietans
Vipera berus
Crotalus cerastes
Bitis atropos
Causus rhombeatus
Atractaspis bibronii
Amplorhinus multimaculatus
Crotaphopeltis hotmaboeia
Dispholidus typus
Rhamphiophis rostratus
Psammophylax rhombeatus
Thelotornis capensis
Psammophis notosticus

Figure 4B

Click here to download Figure Slender ratio.tif

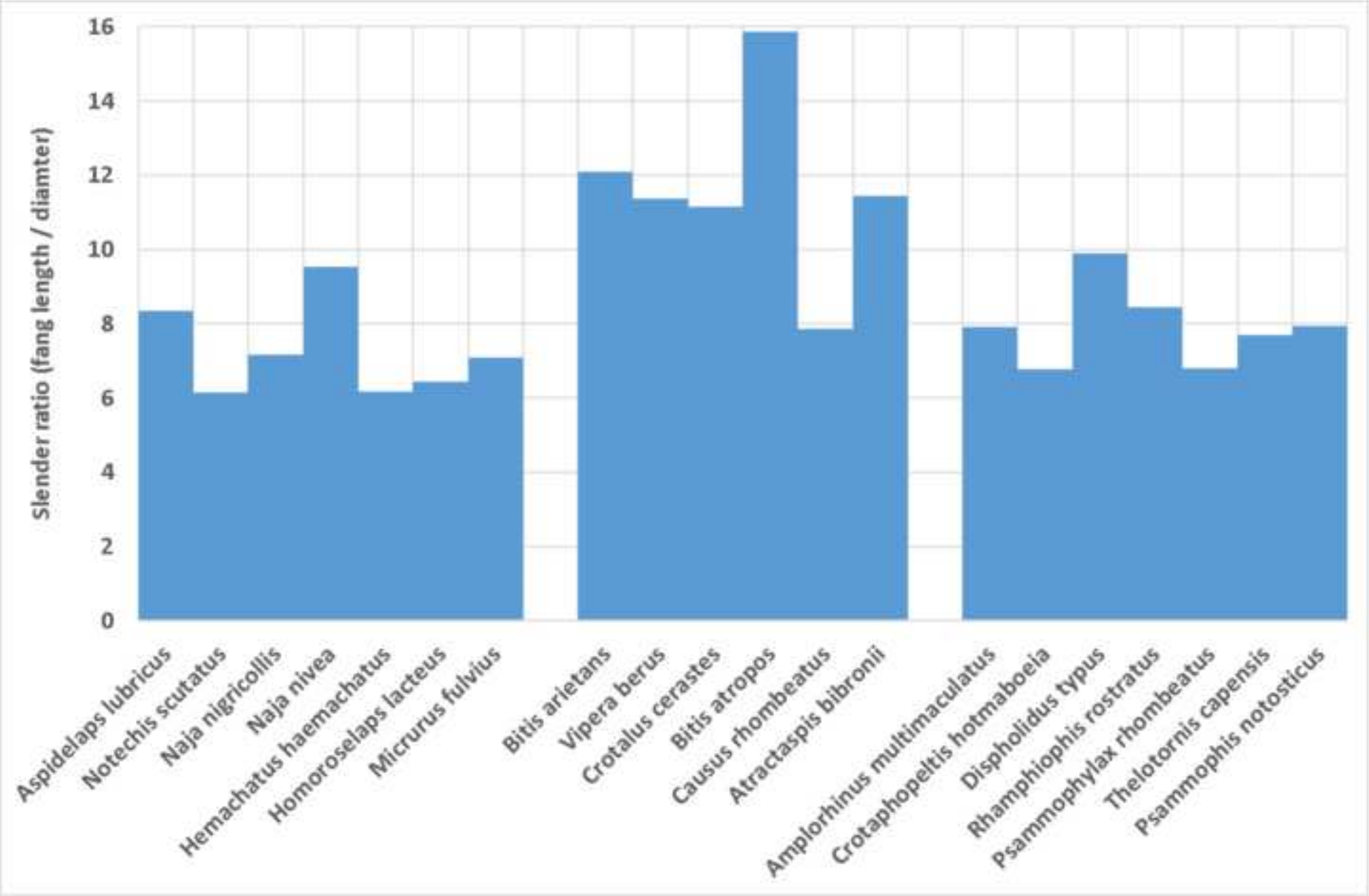

Figure 4C

Click here to download Figure wall thickness.tif

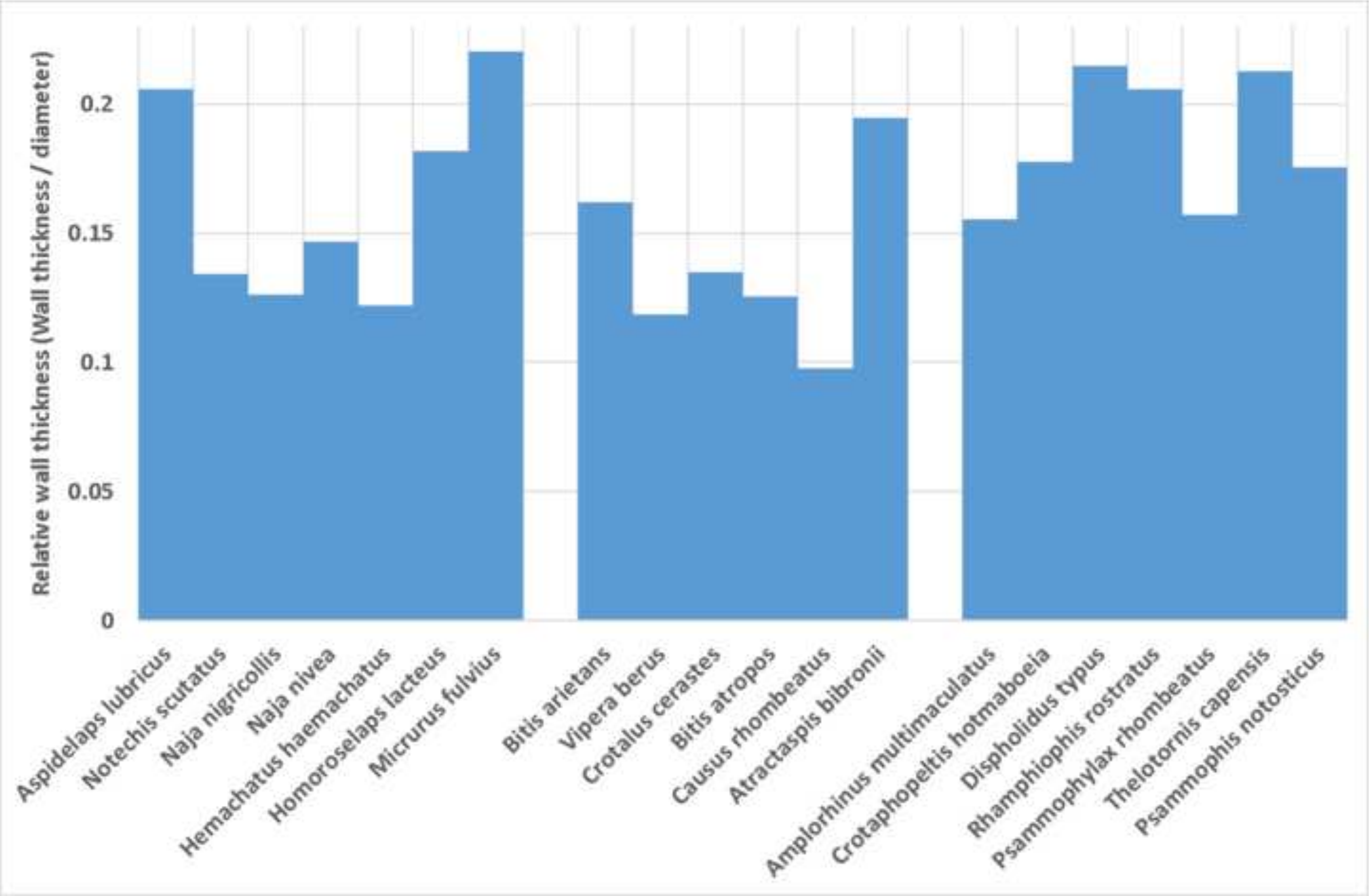

Figure 4D

Click here to download Figure material volume fraction.tif

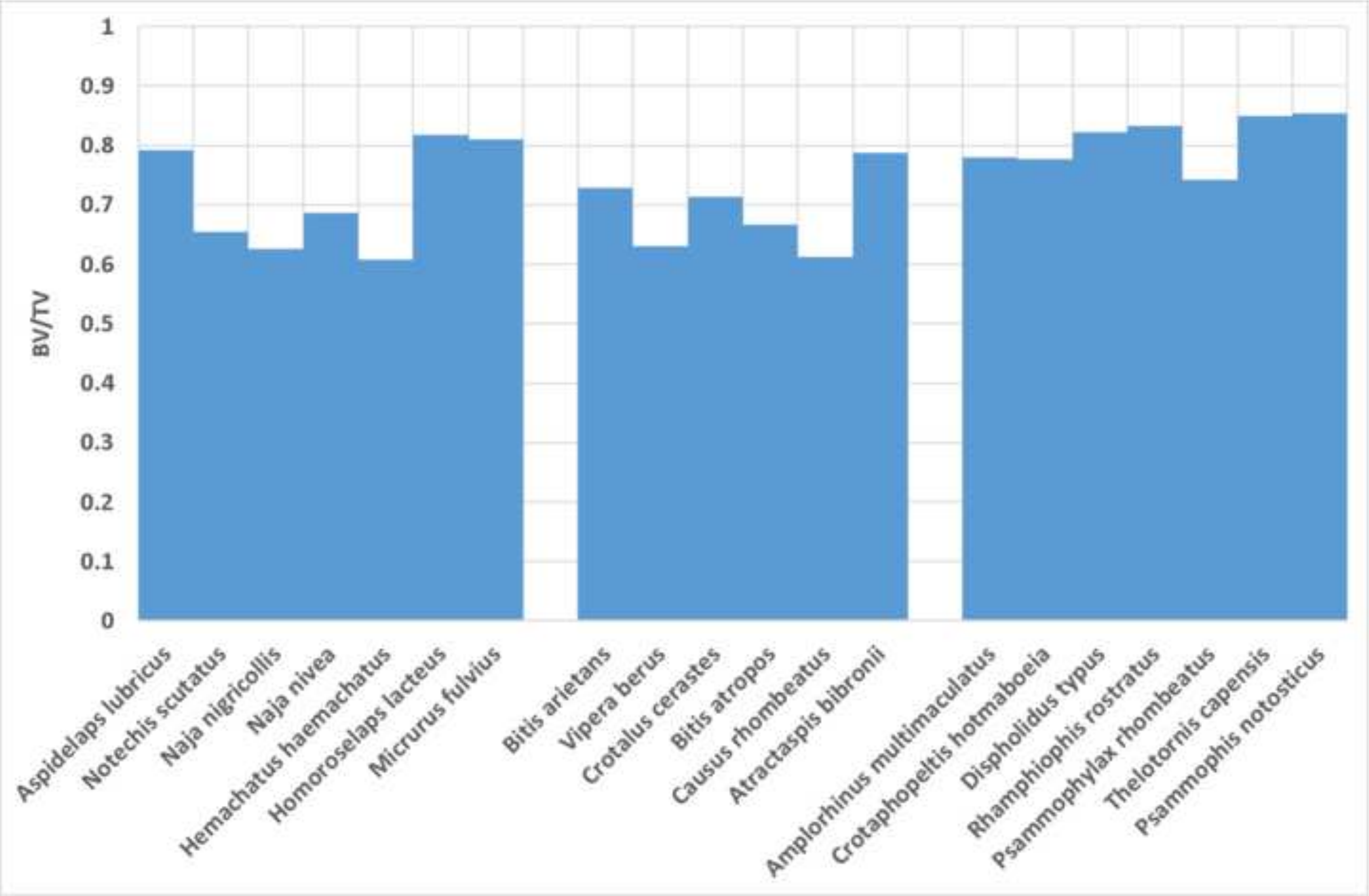

Figure 4E

Click here to download Figure curvature.tif

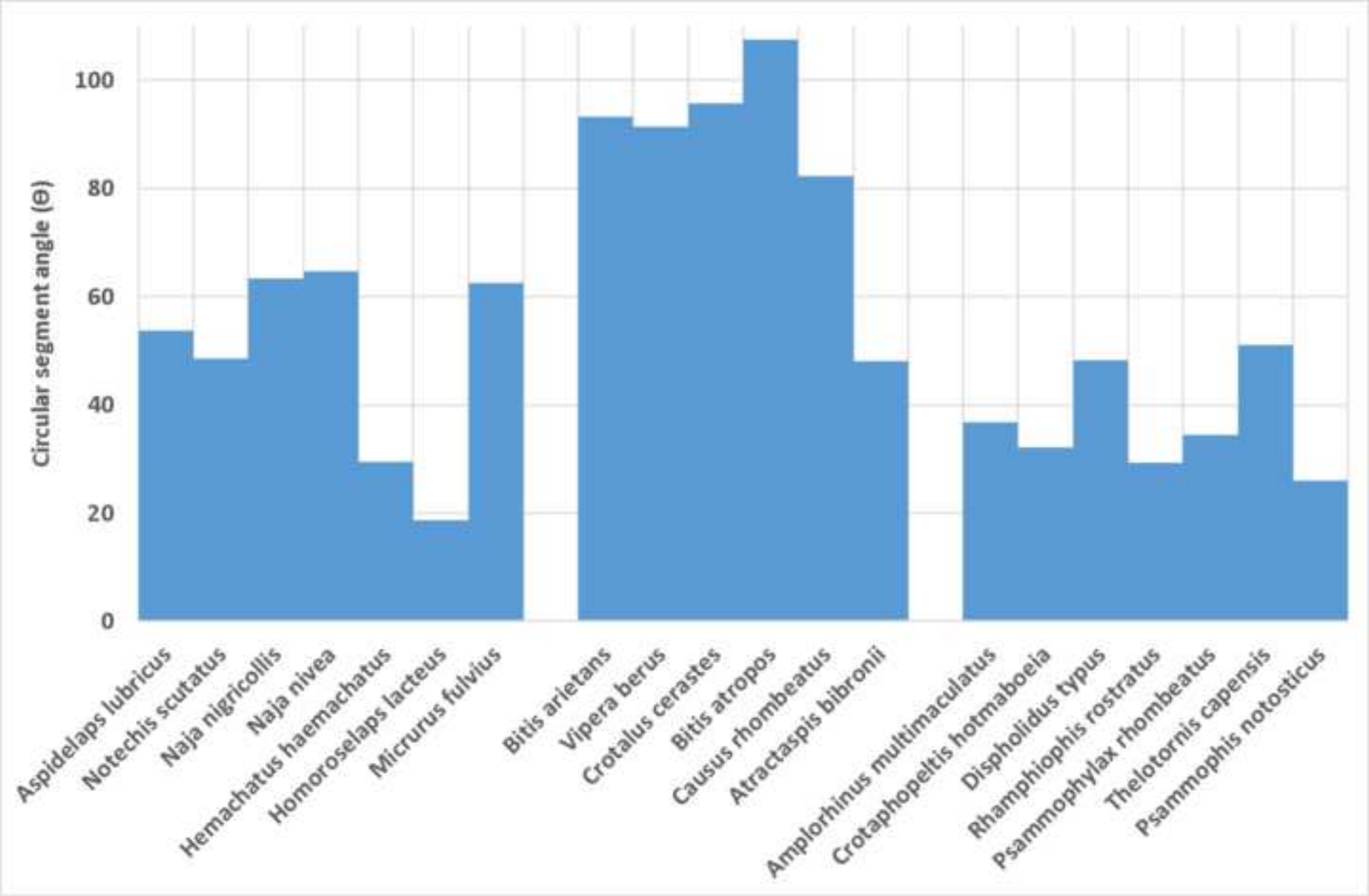

Figure 5

Click here to download Figure Figure 5.tif

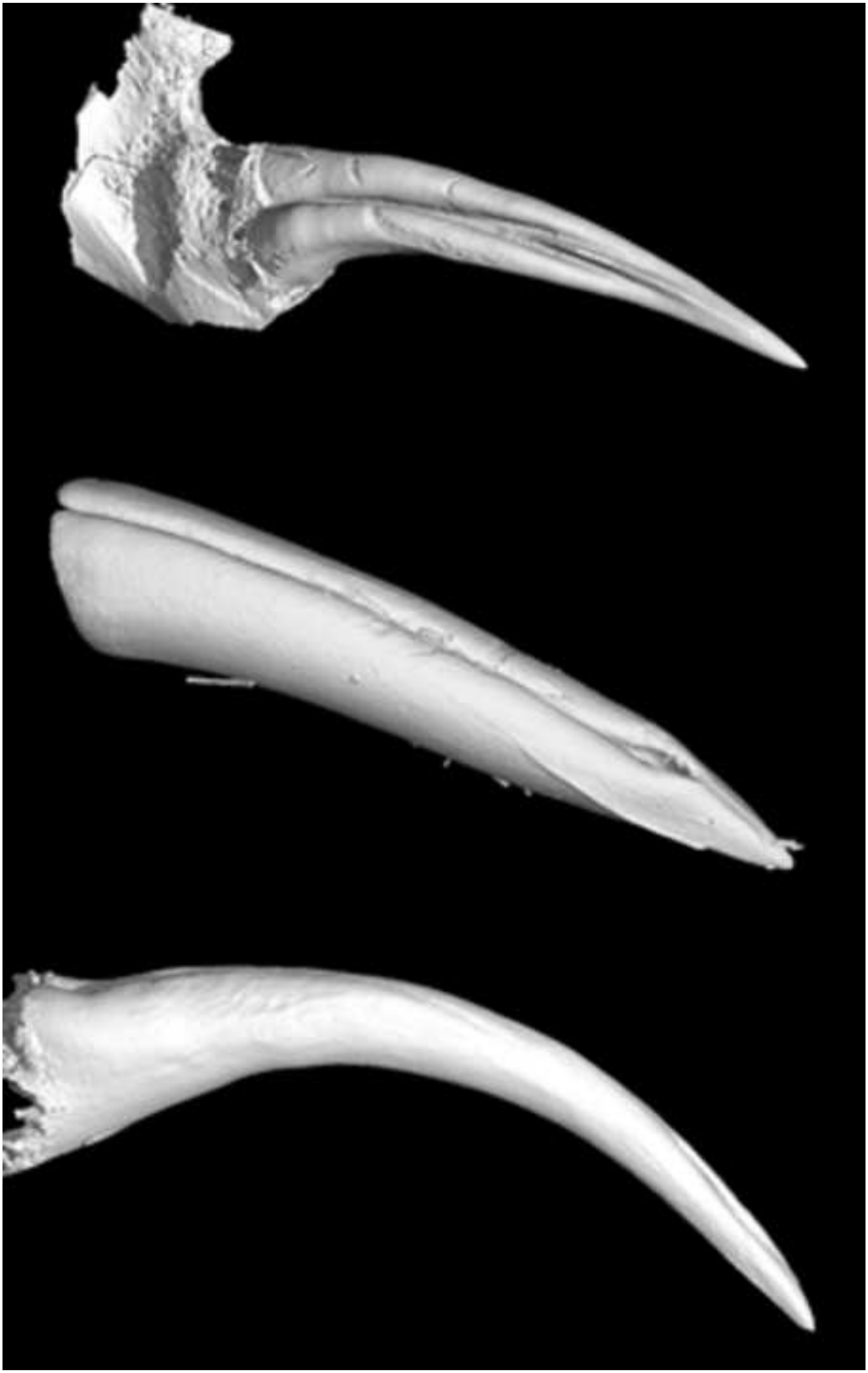

Figure 6A

Click here to download Figure Figure 6a.tif

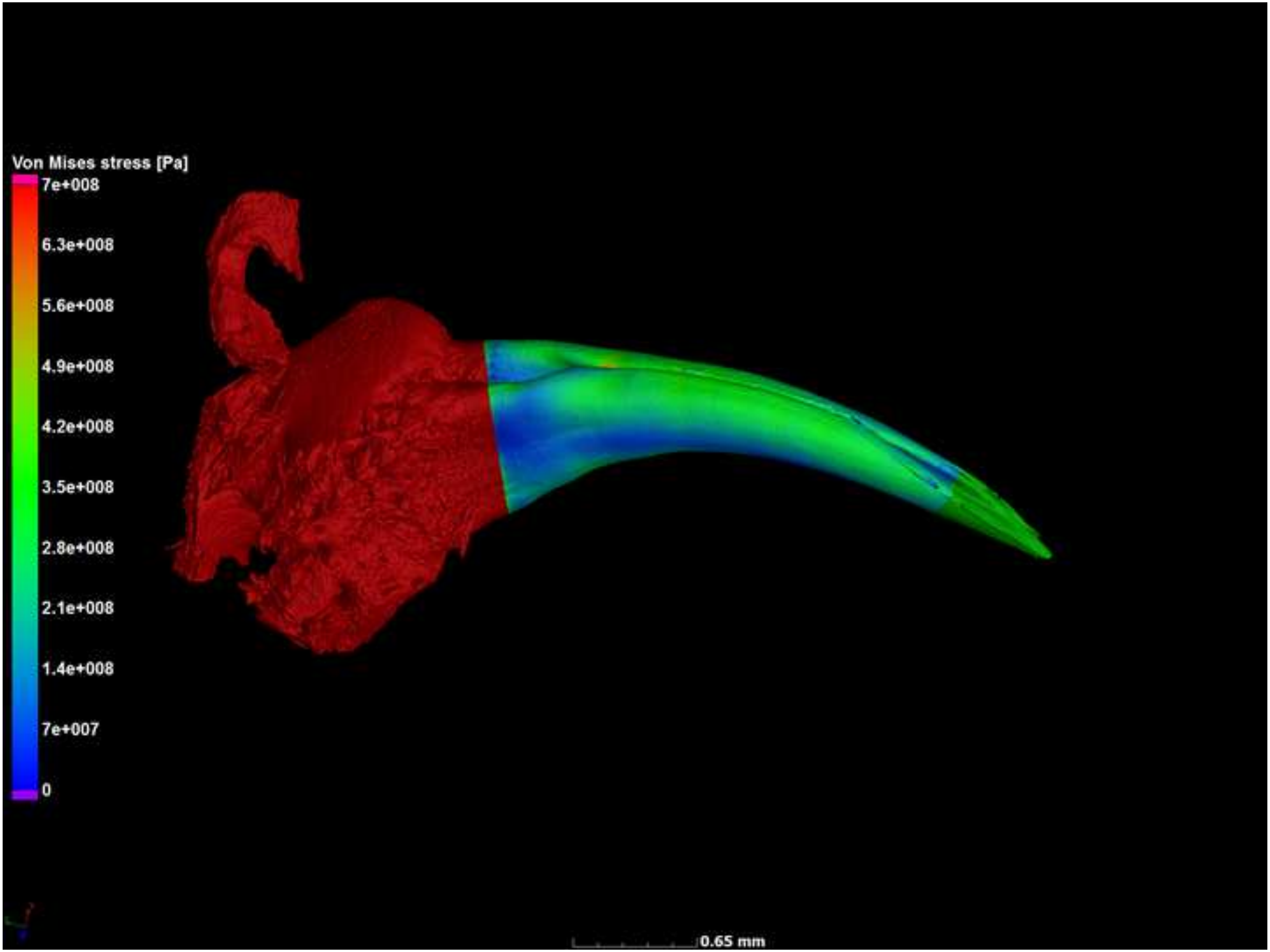

Figure 6B

Click here to download Figure Figure 6b.tif

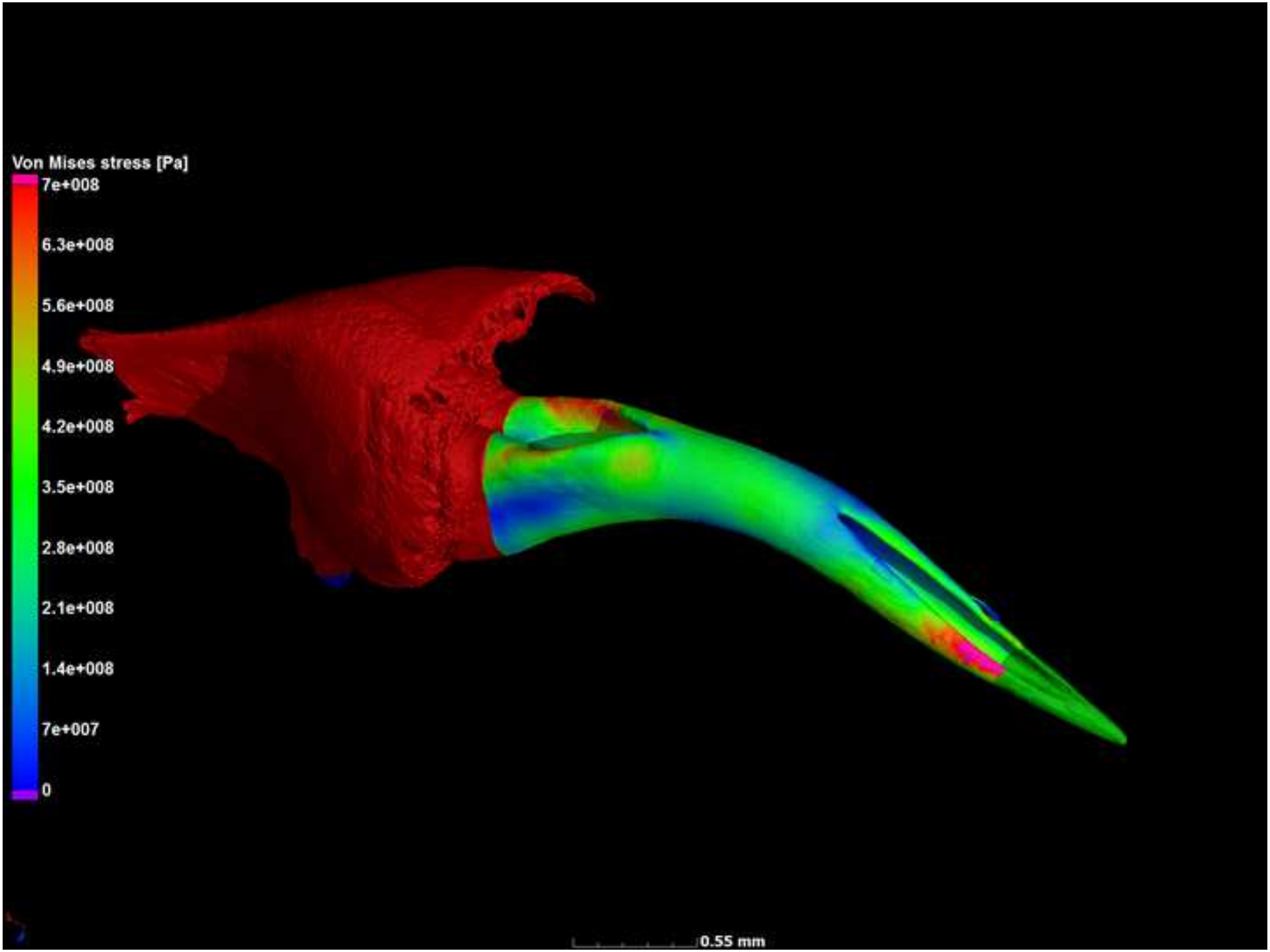

Figure 6C

Click here to download Figure Figure 6c.tif

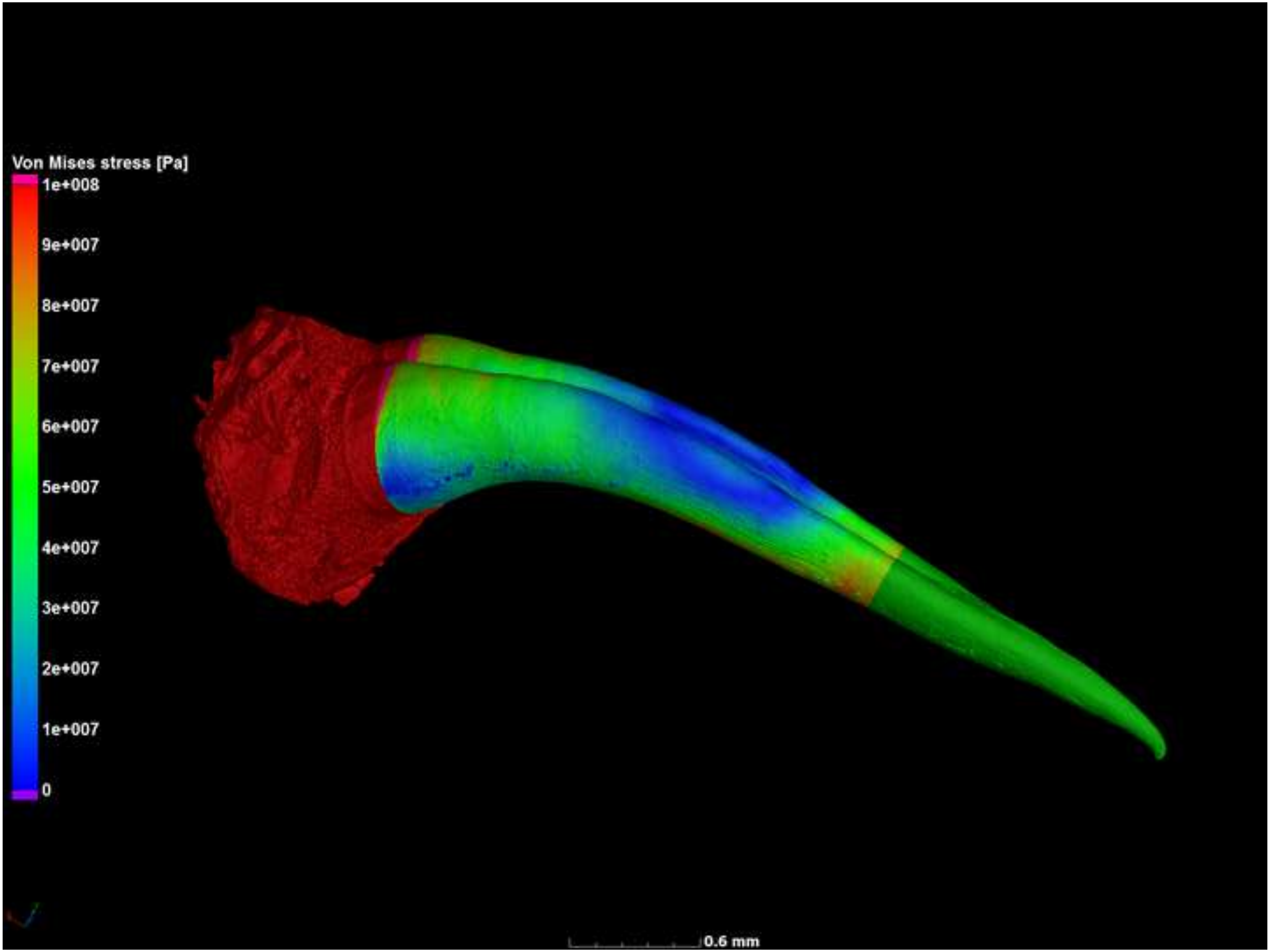

Figure 7A

Click here to download Figure Figure 7a.tif

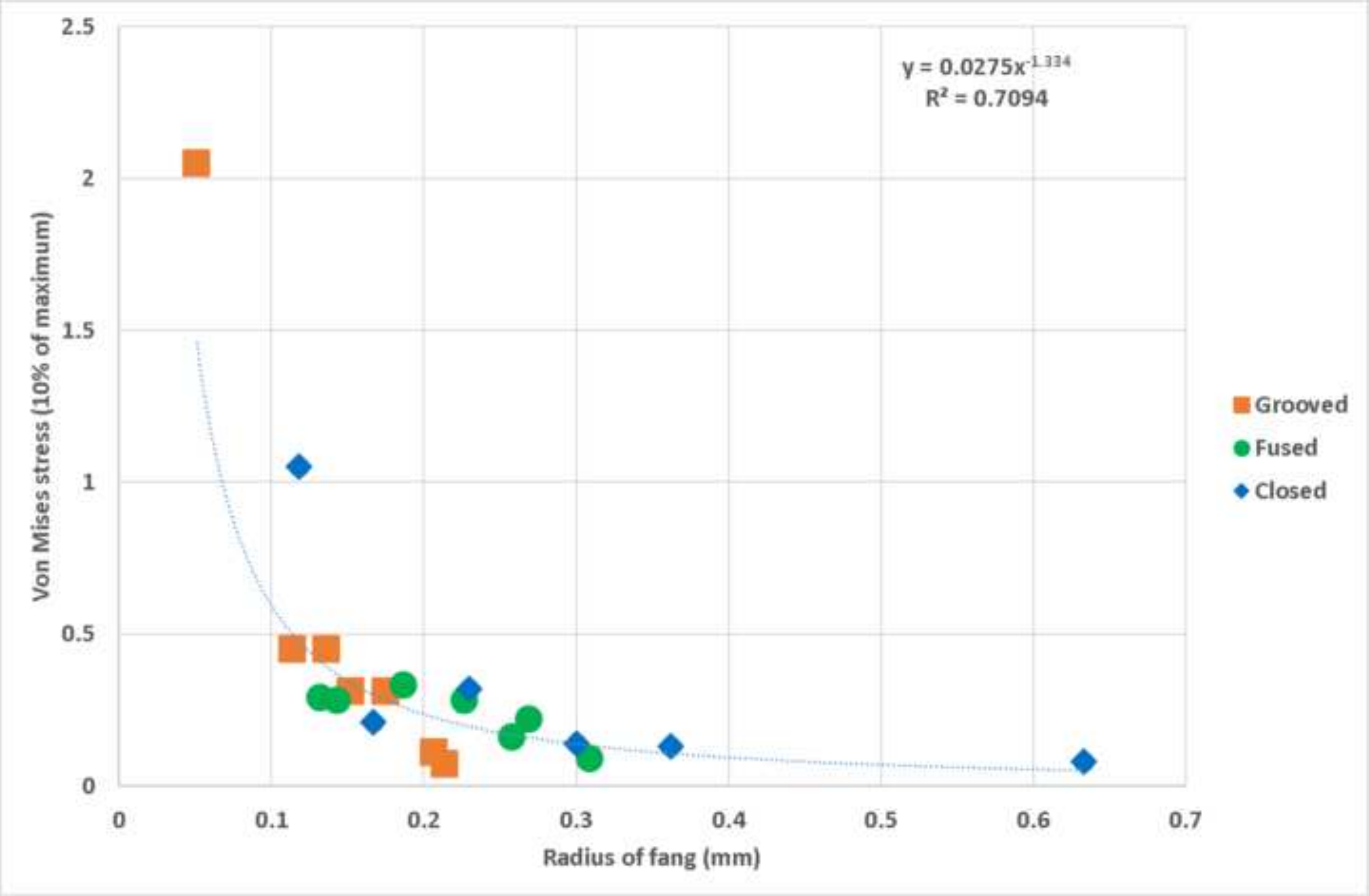

Figure 7B

Click here to download Figure Figure 7b.tif

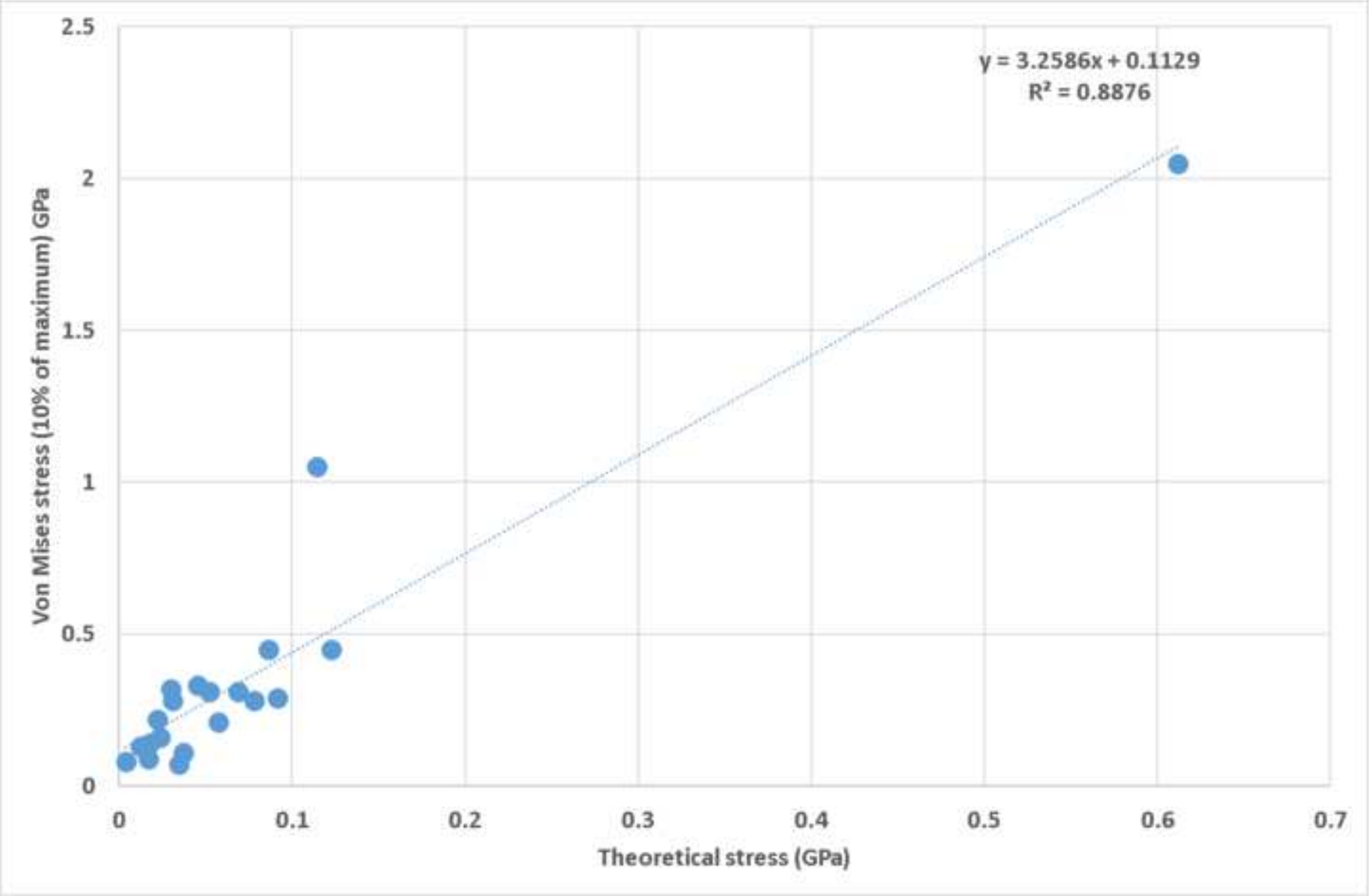

Click here to download Figure Fig 7c.tif

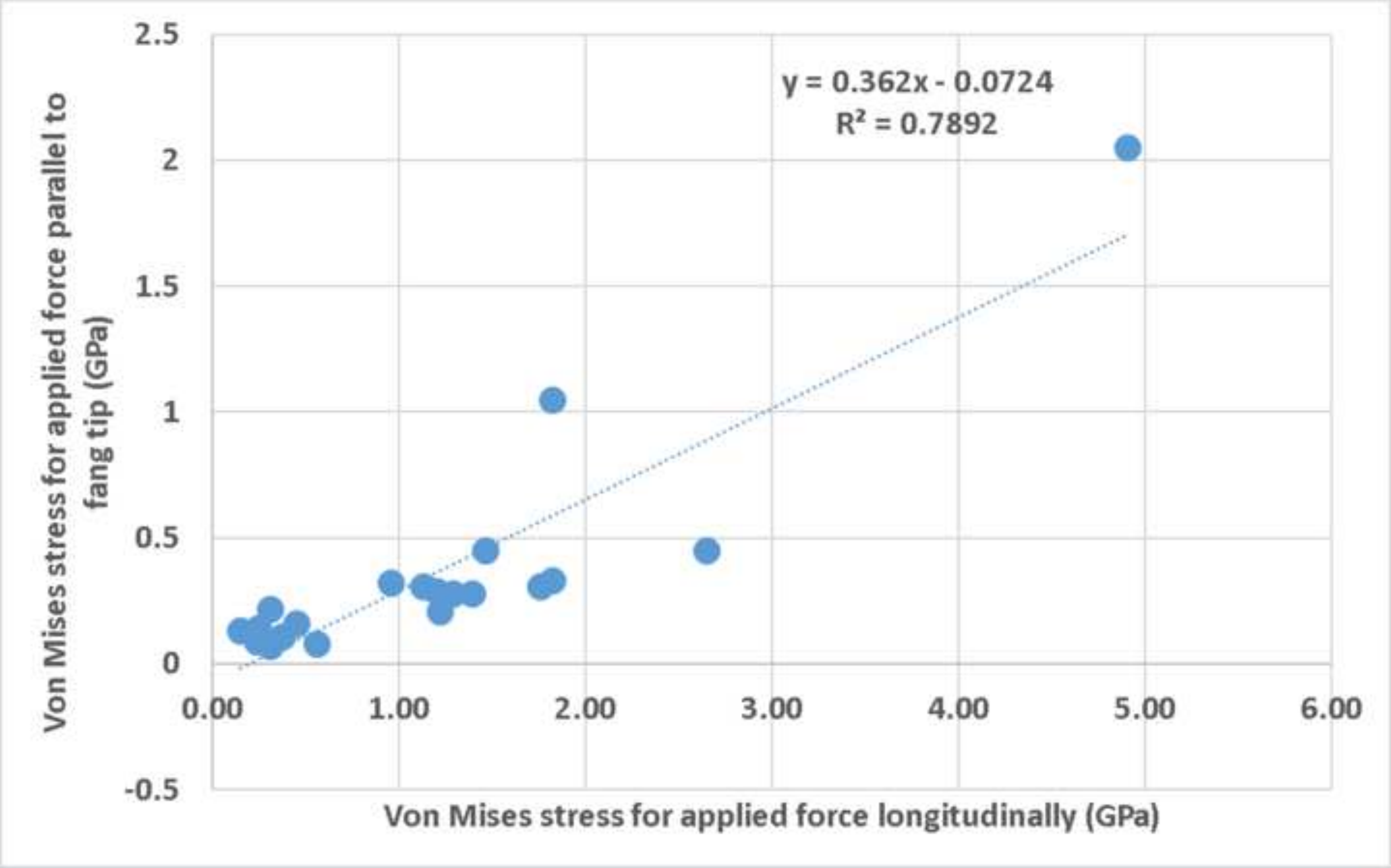

Figure 8

Click here to download Figure Figure 8a.tif

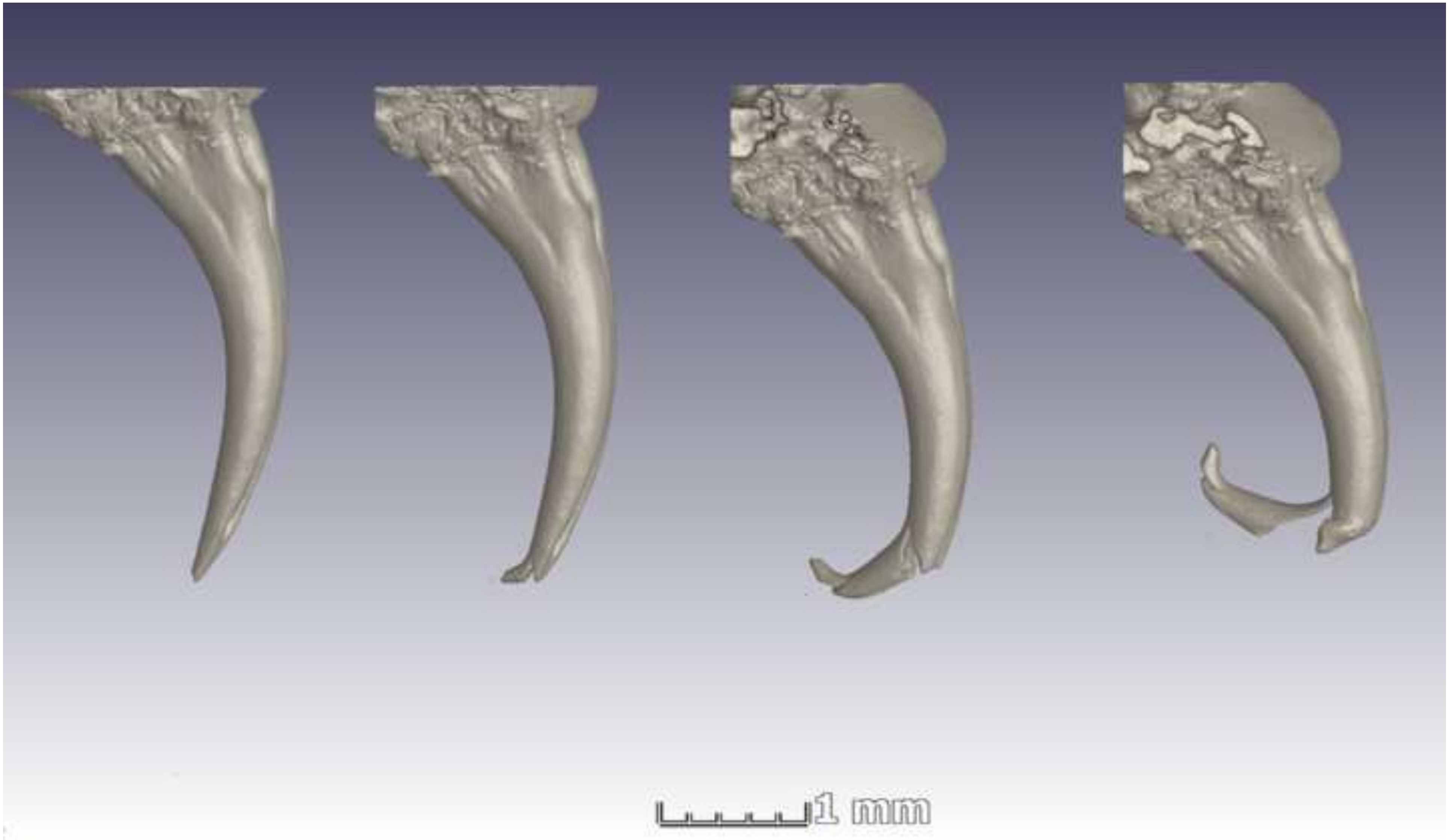

 Click here to download Figure Figure 9.tif

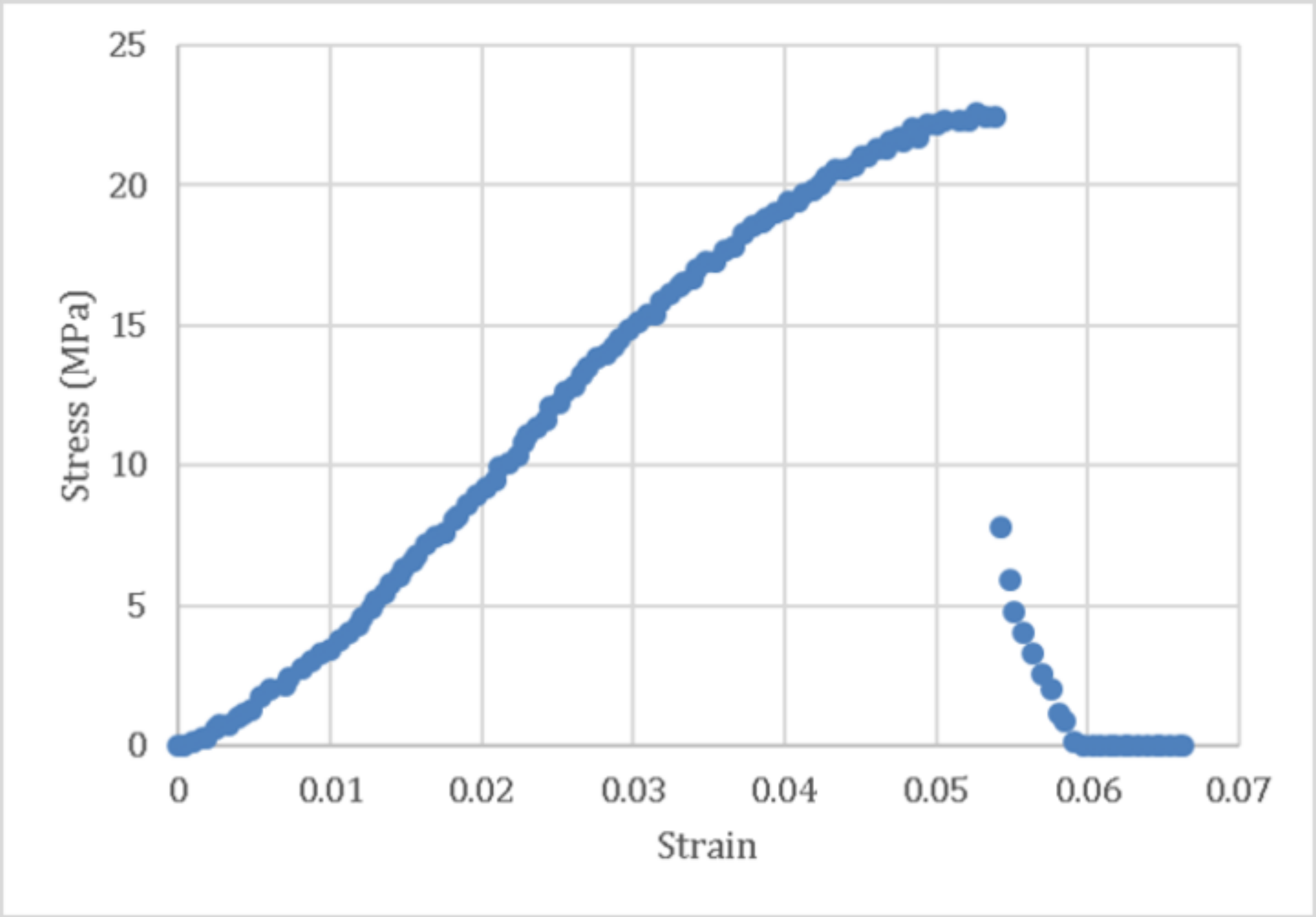

Supplement: GIGA-D-17-00198_Original_Submission.pdf [file gix126_giga-d-17-00198_original_submission.pdf]
