# Supplementary material for: Snake fangs: 3D morphological and mechanical analysis by microCT, simulation, and physical compression testing
Source: Gigascience. 2017 Dec 15;7(1):gix126. doi: 10.1093/gigascience/gix126 (PMC5765556; doi:10.1093/gigascience/gix126)
Supplement: Supplemental material [file gix126_supp.docx]

**Supplementary material: voxel-based simulation applied to real and artificial microCT data**

Voxel-based structural mechanics simulation using VGStudioMax 3.1 was applied to the *Causus rhombeatus* fang as demonstration, mainly to emphasise the fact that the simulation is applied on segmented binary data rather than depending on local grey values. Once the data is segmented, a surface determination is performed. This surface determination is used in the structural mechanics simulation when applied to voxel data and the algorithm used is an immersed-boundary FEM code. Since only a segmentation is required and not a conforming mesh, as is usually required for typical FEM solvers, the method is said to not require a mesh. However, the surface determination is also used directly when generating the mesh (STL file) as is done in this paper for data simplification. Therefore, the STL file can be used if necessary, to create an artificial microCT data set if the original grey values are not present, and the same simulations can be performed. The result is identical, as long as the simulation cell size is the same. This was applied to the *Causus rhombeatus* fang and images below show the identical results and serve to explain this concept. The result for 10% of maximum stress was 88 MPa in both cases.


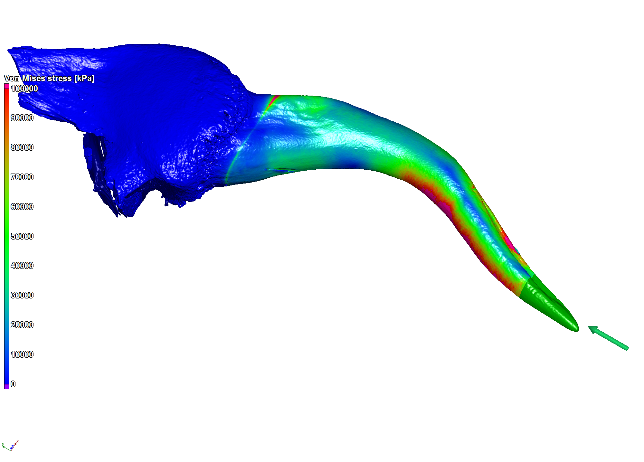

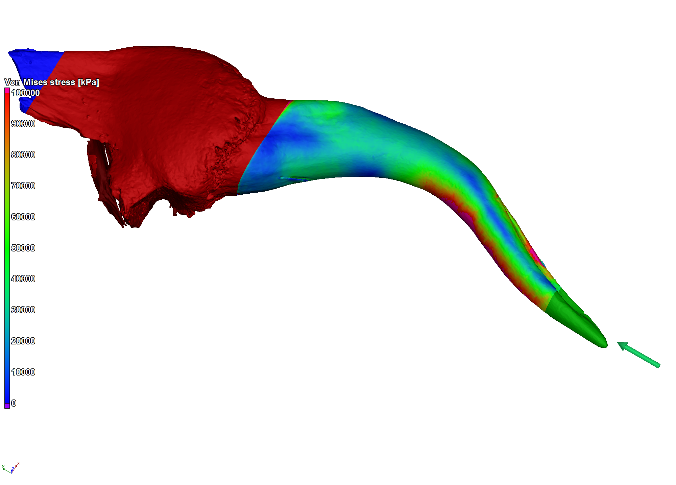


Figure S1: 3D von Mises stress distribution result for (a) microCT voxel data, and (b) artificial voxel data from mesh


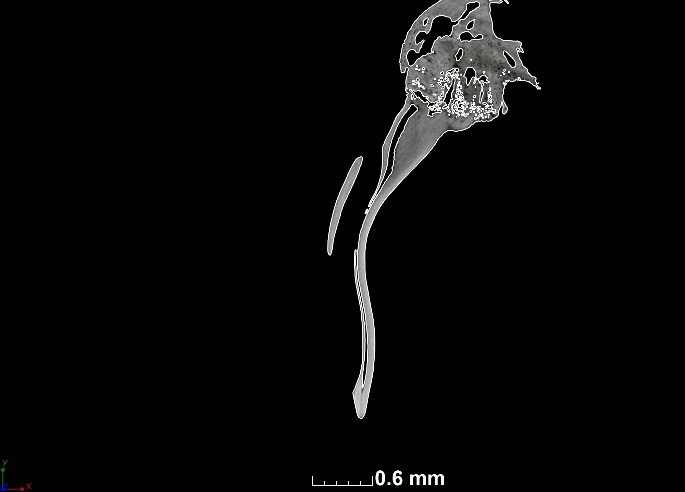

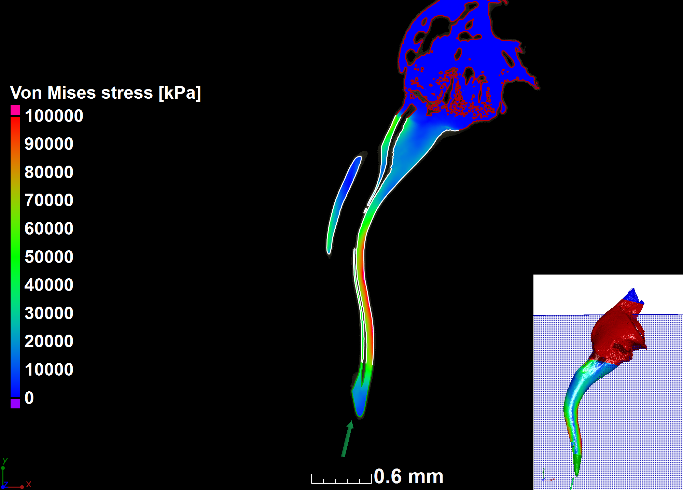


Figure S2: Slice images showing (a) microCT data and (b) von Mises stress distribution


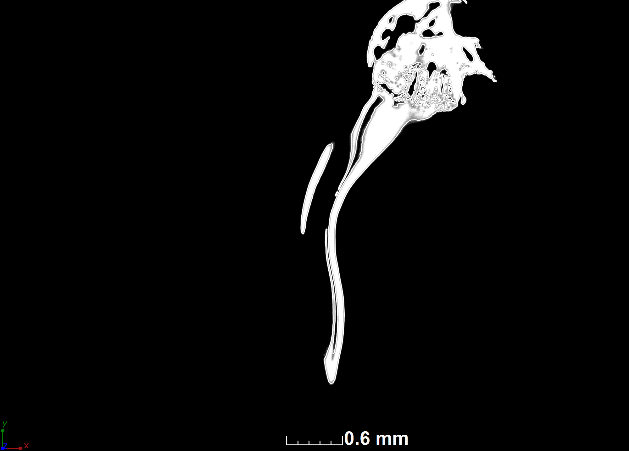

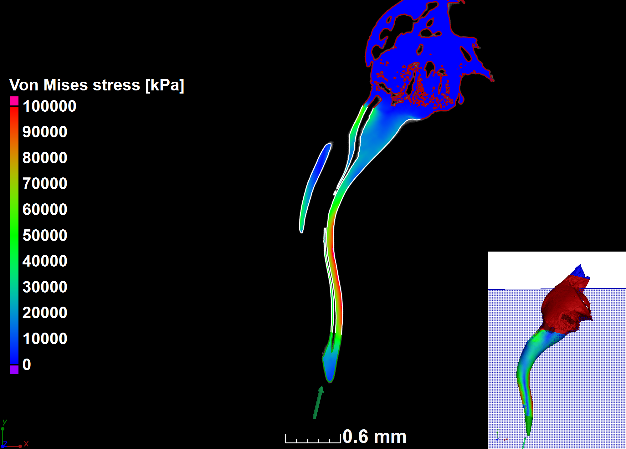


Figure S3: Slice images showing (a) artificial voxel data and (b) von Mises stress distribution


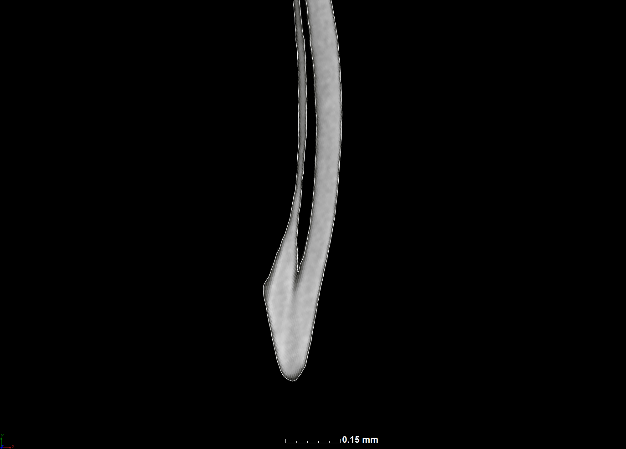

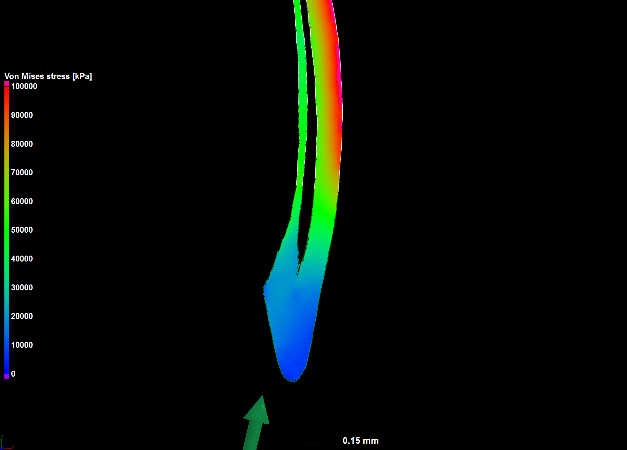


Figure S4: Close-up view of tip of fang for microCT data (a) without and (b) with stress distribution


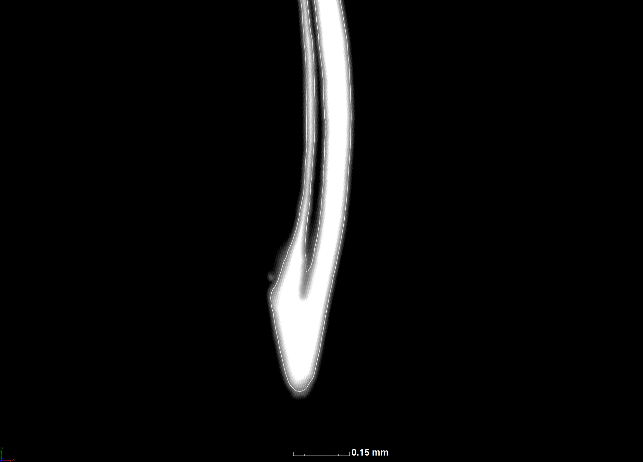

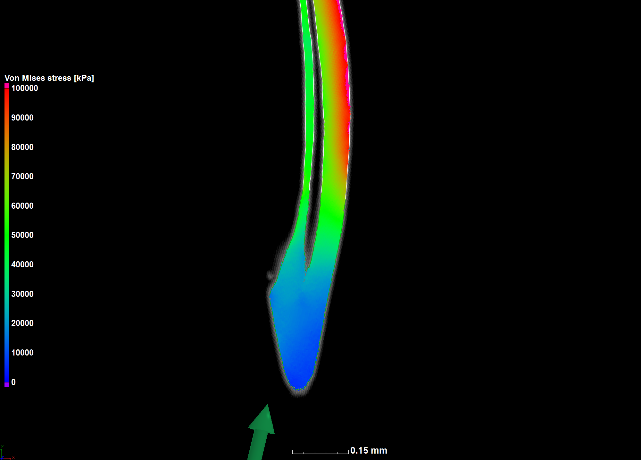


Figure S5: Close-up view of tip of fang for artificial voxel data from mesh (a) without and (b) with stress distribution.
