# Supplementary material for: Snake fangs: 3D morphological and mechanical analysis by microCT, simulation, and physical compression testing
Source: Gigascience. 2017 Dec 15;7(1):gix126. doi: 10.1093/gigascience/gix126 (PMC5765556; doi:10.1093/gigascience/gix126)
Supplement: Response_to_Reviewer_Comments_Original_Submission.pdf [file gix126_response_to_reviewer_comments_original_submission.pdf]

## Author's Response To Reviewer Comments

Reply to reviewer comments

Dear editor,

We have used the comments from the reviewers to improve the manuscript, our replies to each comment is given below in italics and changes in the manuscript are shown using track changes. We have addressed all comments and hope to see the work published soon.

Please thank the reviewers for their comments and time in this process

Regards
-----------------

Reviewer reports:
Reviewer #1: This is a well-written manuscript that describes three snake fang phenotypes and offers a preliminary attempt at correlating species-specific morphological differences with stress distributions from load simulations. The authors make the original CT volumetric data (16-bit TIFF format) publicly available along with surface-rendered 3D image (STL format) derived from these images. The provision of the original CT volumetric data enables researchers to repeat the analysis of voxel-based load simulation. Whereas the data outlined in many of the figures is not statistically significant due to the inherent low number of samples, the approach is novel and the authors are to be commended for this.

Major comments

In the Abstract, the authors state that the "Physical compression results of individual fangs were also used to calculate the effective elastic modulus of the entire snake fang for the first time." However, elastic modulus values of snake fangs have been described previously in key species such as Bitis arietans and Naja nivea (Jansen van Vuuren et al., 2016, J. Raman Spectrosc., 47: 787-795. doi: 10.1002/jrs.4903). The authors should amend this statement.

The values obtained by Jansen van Vuuren et al were obtained using a local indentation method; we are referring here to the effective young’s modulus of the entire structure including the cavities, much like a porous material has a lower young’s modulus compared to a solid of the same material. Despite the low value due to cavities, indications are that the material itself has a lower modulus than that found by indentation, discussed near the end of the paper. This is definitely a novel aspect of the work, which we have now elaborated on in the introduction and discussion. We modify the statement specifically refered to in this comment, as such:

Physical compression results of individual fangs were also used to calculate the effective elastic modulus of the entire snake fang structure including internal cavities for the first time. This elastic modulus of the entire fang is significantly lower than the locally-measured values previously reported from indentation experiments.

In the Conclusions the authors state that "Physical compression tests were conducted for the first time on a snake fang." However, the authors have previously described physical compression tests in a single Bitis sp. snake fang in Figure S2 of a recent publication (Broeckhoven & du Plessis, 2017, Biology Letters Aug;13(8). pii: 20170293. doi: 10.1098/rsbl.2017.0293). The authors should refer to this recent article and should explain how the current study differs from this analysis.

We have changed it. This data note is meant to make available, highlight and detail the data set in the paper refered to above. As such, the analysis methods are described in detail, the data is provided for re-use and the results are discussed in more detail, we modify a paragraph in the Introduction to clarify this:

This data note is meant to highlight this exceptional dataset, providing details on the analysis and providing additional results not included in the original paper. This includes advanced morphological comparisons, more detailed load simulation results and physical compression test data and extraction of elastic modulus values.

The figures and figure legends require more detail. In Figure 4, the number of samples per species used for this analysis should be clearly stated in either the figure or the figure legend.

We add this in the caption: (one sample per species)
And we added more at other captions also

Figure 9 does not detail the species that is described by the stress-strain curve. Furthermore, it is unclear whether the supplementary movie of fang compression failure details the same specimen as shown in Figure 9. In addition, it is unclear whether the specimen shown in the movie of fang compression failure is the same specimen that is shown in Figure 8. More detail in the figure legends would resolve this ambiguity.

On a related note, in the Results and Discussion the authors refer to physical compression testing of two snake fangs. Although not statistically significant, this is a step towards exploring variation between specimens. However, only one of these stress-strain curves is shown in Figure 9. Figure 9 should be extended to include the stress-strain curves of both snake fangs.

We have included more detail in the captions and the two compression tests were both recorded and are now shown in Figure 9, and both live X-ray videos are included as supplementary. The initial idea was to only show one to simplify the discussion. Figure 8 was a different fang of the same type, compressed further to show more failure and allow scanning at each failure point.

Three of the figures appear to show data that have been published previously in a recent publication (Broeckhoven & du Plessis, 2017, Biology Letters Aug;13(8). pii: 20170293. doi: 10.1098/rsbl.2017.0293). Specifically, Figures 3 and 6 in the submitted manuscript look very similar to Figure 1 in the Broeckhoven & du Plessis (2017) paper.

Yes, this is a data note meant to highlight and extend on the previous paper. Figure 3 is meant to

illustrate the three phenotypes and Figure 6 shows typical stress distribution results from load simulations, including videos which were not in the previous paper.

Likewise, Figure 7a, which details the Von Mises stress values in Grooved (n=7), Fused (n=7), and Closed (n=6) phenotypes looks similar to the data previously published in Table 1 of the Broeckhoven & du Plessis (2017) paper. If these data have been published previously, the authors should clearly refer to this in the figure legend.

Figure 7a has been published in a different form in the previous paper, in this figure, the power law is illustrated showing clearly how all three phenotypes fall on the same curve. The data is presented in a different way but we add again the reference to the previous work in the caption as suggested.

Minor comments

Page 2 line 48
The authors should consider revising the phrase "Each fang was individually loading…"

Thanks this is fixed

Page 3 line 84-86
"The series of images in Figure 1 shows the night adder (Causus rhombeatus), with whole-head microCT scan (skin view followed by transparent view showing upper jawbone and skull, then rotated jawbone with circles indicating the location of fangs (including replacement fangs) in mobile anterior position."
This opening sentence to the Results and Discussion section is confusing. The authors should consider revising this sentence to improve clarity.

We changed it

There are type errors in the Supplementary readme.txt file, including:
"Microrurus" - should be "Micrurus"
This was fixed, it was microrus, now corrected.

"simulatio" - should be "simulation"

This was correct, we could not find “simulatio” in the document

Reviewer #2: This manuscript is written as a companion piece to a very recently published paper in Biology Letters entitled "Has snake fang evolution lost its bite? New insights from a structural mechanics viewpoint", expanding on some of the analyses that were performed there. The majority of the methods, findings and discussions already exist in some form and it is not entirely clear which results are new. As such this entire manuscript may have been more

appropriately submitted as supplemental data for the original manuscript. If it is to serve as the authors intended, a clearer summary of the original paper with a detailed outline of the additional approaches included, and how they help expand the findings of the previous study.

This is meant as a data note, to encourage re-use of the data, and provide additional analyses and results not included in the original manuscript. It was meant to supplement and highlight the original work. As mentioned above, this was clarified in the Introduction as such:

This data note is meant to highlight this exceptional dataset, providing details on the analysis and providing additional results not included in the original paper. This includes advanced morphological comparisons, more detailed load simulation results and physical compression test data and extraction of elastic modulus values.

If we are to treat this as an independent manuscript, the paper needs a lot of additional work: The hypotheses are not clearly laid out in the introduction, which simply states that the aim of the paper is to "expand on the findings of the previous analysis" and to "explore if fangs are biomechanically optimized". At no point do the authors outline discuss the evolutionary framework of the fangs, the convergence between phenotype, etc.

It is meant as data note and above paragraph now more clearly states that. The aspects in this paper that are not presented in the previous work are more clearly described now, see comments at reviewer 1 as well and track changes in manuscript.

The authors offer STL files to reanalyze the data, but then explain that their analyses in VolumeGraphicd make use of voxel data, which requires volumetric datasets, not shape files.

We provide full microCT raw data, as well as the STL files. The STL files are the result of the segmentation work, and are the basis of the voxel-based simulation. You could take the STL file, create a voxel data set from it, for example using "convert to volume" in VGStudioMax, and run the simulations. Or you could make new segmentations from the data.

We added in the abstract "the data is provided in the form of image stacks .. and STL files …"

Frustratingly, the referenced material suggests that the authors have not the deep pool of literature that exists for both snake fang morphology and the variety of biomechanical/finite element analyses, with five of the six cited papers being written by the authors themselves.

This is a data note and therefore no further literature references are required in our opinion. The relevant literature is cited in the original paper which this data note refers to.

I wanted to point out that the authors offered to make the stl files available online, to allow for repeatability of the analyses- however, I think that the structural analysis module that they used on VolumeGraphics requires volumetric data, not shape files. The authors kindly made their dataset available to me to test through a FTP, but I suggest they able to upload the tiff-stacks and metadata files to a CT repository site like morphosource, so that folks could recreate the analyses in exactly the same way that the authors did.

Please see comment above on STL file and convert to volume function, also full stacks provided. The full data is freely available in Gigascience, this is the point of the supporting data note, to describe in more detail and relate the data to the previously published work based on it. It will be very easily accessible in Gigascience, and citable.

I would also like to have a more detailed explanation of the choice of settings in the that the authors used for the structural analysis module: there have not been too many published papers that use this method with this software and I think it would be very useful if they set out, step by step, why they chose the settings they chose (max number of iterations, cell size, etc).

For load simulation, the description of the methodology and settings used is expanded in the Methods section, see track changes in manuscript
