# Supplementary material for: Snake fangs: 3D morphological and mechanical analysis by microCT, simulation, and physical compression testing
Source: Gigascience. 2017 Dec 15;7(1):gix126. doi: 10.1093/gigascience/gix126 (PMC5765556; doi:10.1093/gigascience/gix126)
Supplement: Response_to_Reviewer_Comments_Revision_1.pdf [file gix126_response_to_reviewer_comments_revision_1.pdf]

Author's Response To Reviewer Comments

Dear editor,

Here our replies to reviewer comments on R1, we submit R2 with track changes and point by point replies below in italics

Hope to hear from you soon, and if you are in need of a journal cover image the new Figure S1 is a good potential option

regards

Reviewer reports:
Reviewer #1: The authors have addressed my comments. Importantly, they have explained how measures of the effective elastic modulus of the entire snake fang are an improvement on previous indentation studies of snake fangs. The mechanical simulation data is supported by data obtained from real-life physical compression tests, and highlights an important method of correlating 3D image data with mechanical models. The stress distributions are presented both visually and quantitatively, and the image quality of the microCT dataset is excellent. The authors are to be commended for making this dataset publicly available.

I've a few comments to add to Reviewer 2's suggestions.

Reviewer 2 suggests archiving the CT images in MorphoSource. MorphoSource enables stacks of TIFF or DICOM images to be uploaded as ZIP format archives, with a preview image randomly extracted from the stack for presentation. GigaDB is an excellent database resource for this type of data as it allows image stacks to be downloaded, but additionally allows users to interactively explore surface-rendered 3D reconstructions prior to data download. I would recommend archiving the CT images in GigaDB.

With respect to the analysis, Reviewer 2 makes a good point in that the authors:
"…offer STL files to reanalyze the data, but then explain that their analyses in VolumeGraphic make use of voxel data, which requires volumetric datasets, not shape files."

The authors response to this is that:
"You could take the STL file, create a voxel data set from it, for example using "convert to volume" in VGStudioMax, and run the simulations."

I feel this response is unsatisfactory. The method that is outlined in the paper utilizes the Structural Mechanics Simulation Module of the commercial software package VGStudioMax. This Module simulates mechanical stress directly on voxel data, and reportedly eliminates meshing as a source of error compared to established methods (https://www.volumegraphics.com/en/news/volume-graphics-releases-structural-mechanics-simulation-for-vgstudio-max-3-0.html). I would envisage that a simulation that starts by

converting mesh data to voxel would be different from a simulation that uses voxel data directly. I would invite the authors to clarify on this point. Furthermore, if the authors have compared how similar the two approaches are, then I would ask them to submit their findings in a supplementary file.

Our reply was based on the fact that the simplified form of the STL file, which makes easy handling of the data possible, COULD ALSO be used. Of course a direct comparison should be done with the original voxel data – however we envisage most researchers to want to use the already-segmented STL data and make new types of simulations, in most cases in other softwares. We have made it clear that we also provide the voxel data, which can be used as explained to make voxel-based simulations, after proper segmentation has been performed.

This comment however points out one important thing to highlight for readers of this article: the voxel-based simulation method does not use greyscale values, but is crucially dependant on the segmentation – ie. it uses effectively a binary segmentation based on the surface determination (delineation between material and air). This is why taking an STL file, making a voxel data set and applying the simulation function will provide the same result – if the same simulation cell size is used. We explain this more clearly and add the suggested simulation comparison as supplementary material, it will help to explain the concepts.

This is what is added in the text using track changes:
The simulation requires a binary segmentation in the form of a surface determination, but does not require a mesh. Nevertheless, a mesh is also generated for simple data handling, and can in principle also be used for simulation, with appropriate remeshing or creation of artificial voxel data based on the mesh file – this is discussed in more detail in the supplementary material
