# Supplementary material for: Snake fangs: 3D morphological and mechanical analysis by microCT, simulation, and physical compression testing
Source: Gigascience. 2017 Dec 15;7(1):gix126. doi: 10.1093/gigascience/gix126 (PMC5765556; doi:10.1093/gigascience/gix126)
Supplement: Reviewer_1_Report_(Original_Submission) -- Chris Armit — 31 Aug 2017 Reviewed [file gix126_reviewer_1_report_(original_submission).pdf]

Reviewer Report

Title: Snake fangs: 3D morphological and mechanical analysis by microCT, simulation and physical compression testing

Version: Original Submission **Date:** 8/31/2017

Reviewer name: Chris Armit

Reviewer Comments to Author:

This is a well-written manuscript that describes three snake fang phenotypes and offers a preliminary attempt at correlating species-specific morphological differences with stress distributions from load simulations. The authors make the original CT volumetric data (16-bit TIFF format) publicly available along with surface-rendered 3D image (STL format) derived from these images. The provision of the original CT volumetric data enables researchers to repeat the analysis of voxel-based load simulation. Whereas the data outlined in many of the figures is not statistically significant due to the inherent low number of samples, the approach is novel and the authors are to be commended for this.Major commentsIn the Abstract, the authors state that the "Physical compression results of individual fangs were also used to calculate the effective elastic modulus of the entire snake fang for the first time." However, elastic modulus values of snake fangs have been described previously in key species such as Bitis arietans and Naja nivea (Jansen van Vuuren et al., 2016, J. Raman Spectrosc., 47: 787-795. doi: 10.1002/jrs.4903). The authors should amend this statement.In the Conclusions the authors state that "Physical compression tests were conducted for the first time on a snake fang." However, the authors have previously described physical compression tests in a single Bitis sp. snake fang in Figure S2 of a recent publication (Broeckhoven & du Plessis, 2017, Biology Letters Aug;13(8). pii: 20170293. doi: 10.1098/rsbl.2017.0293). The authors should refer to this recent article and should explain how the current study differs from this analysis.The figures and figure legends require more detail. In Figure 4, the number of samples per species used for this analysis should be clearly stated in either the figure or the figure legend. Figure 9 does not detail the species that is described by the stress-strain curve. Furthermore, it is unclear whether the supplementary movie of fang compression failure details the same specimen as shown in Figure 9. In addition, it is unclear whether the specimen shown in the movie of fang compression failure is the same specimen that is shown in Figure 8. More detail in the figure legends would resolve this ambiguity.On a related note, in the Results and Discussion the authors refer to physical compression testing of two snake fangs. Although not statistically significant, this is a step towards exploring variation between specimens. However, only one of these stress-strain curves is shown in Figure 9. Figure 9 should be extended to include the stress-strain curves of both snake fangs.Three of the figures appear to show data that have been published previously in a recent publication (Broeckhoven & du Plessis, 2017, Biology Letters Aug;13(8). pii: 20170293. doi: 10.1098/rsbl.2017.0293). Specifically, Figures 3 and 6 in the submitted manuscript look very similar to Figure 1 in the Broeckhoven & du Plessis (2017) paper. Likewise, Figure 7a, which details the Von Mises stress values in Grooved (n=7), Fused (n=7), and Closed (n=6) phenotypes looks similar to the data previously published in Table 1 of the Broeckhoven & du Plessis (2017) paper. If these data have been

published previously, the authors should clearly refer to this in the figure legend.Minor commentsPage 2 line 48The authors should consider revising the phrase "Each fang was individually loading…"Page 3 line 84-86"The series of images in Figure 1 shows the night adder (Causus rhombeatus), with whole-head microCT scan (skin view followed by transparent view showing upper jawbone and skull, then rotated jawbone with circles indicating the location of fangs (including replacement fangs) in mobile anterior position."This opening sentence to the Results and Discussion section is confusing. The authors should consider revising this sentence to improve clarity.There are type errors in the Supplementary readme.txt file, including:"Microrurus" - should be "Micrurus""simulatio" - should be "simulation"

Level of Interest

Please indicate how interesting you found the manuscript: An article whose findings are important to those with closely related research interests

Quality of Written English

Please indicate the quality of language in the manuscript: Acceptable

Declaration of Competing Interests

Please complete a declaration of competing interests, considering the following questions:

- Have you in the past five years received reimbursements, fees, funding, or salary from an organisation that may in any way gain or lose financially from the publication of this manuscript, either now or in the future?
- Do you hold any stocks or shares in an organisation that may in any way gain or lose financially from the publication of this manuscript, either now or in the future?
- Do you hold or are you currently applying for any patents relating to the content of the manuscript?
- Have you received reimbursements, fees, funding, or salary from an organization that holds or has applied for patents relating to the content of the manuscript?
- Do you have any other financial competing interests?
- Do you have any non-financial competing interests in relation to this paper?

If you can answer no to all of the above, write 'I declare that I have no competing interests' below. If your reply is yes to any, please give details below.

I declare that I have no competing interests.

I agree to the open peer review policy of the journal. I understand that my name will be included on my report to the authors and, if the manuscript is accepted for publication, my named report including any attachments I upload will be posted on the website along with the authors' responses. I agree for my

report to be made available under an Open Access Creative Commons CC-BY license (http://creativecommons.org/licenses/by/4.0/). I understand that any comments which I do not wish to be included in my named report can be included as confidential comments to the editors, which will not be published.

I agree to the open peer review policy of the journal
