# Supplementary material for: Snake fangs: 3D morphological and mechanical analysis by microCT, simulation, and physical compression testing
Source: Gigascience. 2017 Dec 15;7(1):gix126. doi: 10.1093/gigascience/gix126 (PMC5765556; doi:10.1093/gigascience/gix126)
Supplement: Reviewer_1_Report_(Revision_1) -- Chris Armit — 22 Nov 2017 Reviewed [file gix126_reviewer_1_report_(revision_1).pdf]

Reviewer Report

Title: Snake fangs: 3D morphological and mechanical analysis by microCT, simulation and physical compression testing

Version: Revision 1 **Date:** 11/22/2017

Reviewer name: Chris Armit

Reviewer Comments to Author:

The authors have addressed my comments. Importantly, they have explained how measures of the effective elastic modulus of the entire snake fang are an improvement on previous indentation studies of snake fangs. The mechanical simulation data is supported by data obtained from real-life physical compression tests, and highlights an important method of correlating 3D image data with mechanical models. The stress distributions are presented both visually and quantitatively, and the image quality of the microCT dataset is excellent. The authors are to be commended for making this dataset publicly available.I've a few comments to add to Reviewer 2's suggestions.Reviewer 2 suggests archiving the CT images in MorphoSource. MorphoSource enables stacks of TIFF or DICOM images to be uploaded as ZIP format archives, with a preview image randomly extracted from the stack for presentation. GigaDB is an excellent database resource for this type of data as it allows image stacks to be downloaded, but additionally allows users to interactively explore surface-rendered 3D reconstructions prior to data download. I would recommend archiving the CT images in GigaDB.With respect to the analysis, Reviewer 2 makes a good point in that the authors:"…offer STL files to reanalyze the data, but then explain that their analyses in VolumeGraphic make use of voxel data, which requires volumetric datasets, not shape files."The authors response to this is that:"You could take the STL file, create a voxel data set from it, for example using "convert to volume" in VGStudioMax, and run the simulations."I feel this response is unsatisfactory. The method that is outlined in the paper utilizes the Structural Mechanics Simulation Module of the commercial software package VGStudioMax. This Module simulates mechanical stress directly on voxel data, and reportedly eliminates meshing as a source of error compared to established methods (https://www.volumegraphics.com/en/news/volume-graphics-releases-structural-mechanics-simulation-for-vgstudio-max-3-0.html). I would envisage that a simulation that starts by converting mesh data to voxel would be different from a simulation that uses voxel data directly. I would invite the authors to clarify on this point. Furthermore, if the authors have compared how similar the two approaches are, then I would ask them to submit their findings in a supplementary file.

Level of Interest

Please indicate how interesting you found the manuscript: An article whose findings are important to those with closely related research interests

Quality of Written English

Please indicate the quality of language in the manuscript: Acceptable

Declaration of Competing Interests

Please complete a declaration of competing interests, considering the following questions:

- Have you in the past five years received reimbursements, fees, funding, or salary from an organisation that may in any way gain or lose financially from the publication of this manuscript, either now or in the future?
- Do you hold any stocks or shares in an organisation that may in any way gain or lose financially from the publication of this manuscript, either now or in the future?
- Do you hold or are you currently applying for any patents relating to the content of the manuscript?
- Have you received reimbursements, fees, funding, or salary from an organization that holds or has applied for patents relating to the content of the manuscript?
- Do you have any other financial competing interests?
- Do you have any non-financial competing interests in relation to this paper?

If you can answer no to all of the above, write 'I declare that I have no competing interests' below. If your reply is yes to any, please give details below.

I declare that I have no competing interests.

I agree to the open peer review policy of the journal. I understand that my name will be included on my report to the authors and, if the manuscript is accepted for publication, my named report including any attachments I upload will be posted on the website along with the authors' responses. I agree for my report to be made available under an Open Access Creative Commons CC-BY license (http://creativecommons.org/licenses/by/4.0/). I understand that any comments which I do not wish to be included in my named report can be included as confidential comments to the editors, which will not be published.

I agree to the open peer review policy of the journal
