# Supplementary material for: Snake fangs: 3D morphological and mechanical analysis by microCT, simulation, and physical compression testing
Source: Gigascience. 2017 Dec 15;7(1):gix126. doi: 10.1093/gigascience/gix126 (PMC5765556; doi:10.1093/gigascience/gix126)
Supplement: Reviewer_2_Report_(Original_Submission) -- Ed Stanley — 17 Nov 2017 Reviewed [file gix126_reviewer_2_report_(original_submission).pdf]

Reviewer Report

Title: Snake fangs: 3D morphological and mechanical analysis by microCT, simulation and physical compression testing

Version: Original Submission **Date:** 11/17/2017

Reviewer name: Ed Stanley

Reviewer Comments to Author:

This manuscript is written as a companion piece to a very recently published paper in Biology Letters entitled "Has snake fang evolution lost its bite? New insights from a structural mechanics viewpoint", expanding on some of the analyses that were performed there. The majority of the methods, findings and discussions already exist in some form and it is not entirely clear which results are new. As such this entire manuscript may have been more appropriately submitted as supplemental data for the original manuscript. If it is to serve as the authors intended, a clearer summary of the original paper with a detailed outline of the additional approaches included, and how they help expand the findings of the previous study.If we are to treat this as an independent manuscript, the paper needs a lot of additional work: The hypotheses are not clearly laid out in the introduction, which simply states that the aim of the paper is to "expand on the findings of the previous analysis" and to "explore if fangs are biomechanically optimized". At no point do the authors outline discuss the evolutionary framework of the fangs, the convergence between phenotype, etc.The authors offer STL files to reanalyze the data, but then explain that their analyses in VolumeGraphicd make use of voxel data, which requires volumetric datasets, not shape files.Frustratingly, the referenced material suggests that the authors have not the deep pool of literature that exists for both snake fang morphology and the variety of biomechanical/finite element analyses, with five of the six cited papers being written by the authors themselves.

Level of Interest

Please indicate how interesting you found the manuscript: An article of limited interest

Quality of Written English

Please indicate the quality of language in the manuscript: Acceptable

Declaration of Competing Interests

Please complete a declaration of competing interests, considering the following questions:

- Have you in the past five years received reimbursements, fees, funding, or salary from an organisation that may in any way gain or lose financially from the publication of this manuscript, either now or in the future?

- Do you hold any stocks or shares in an organisation that may in any way gain or lose financially from the publication of this manuscript, either now or in the future?
- Do you hold or are you currently applying for any patents relating to the content of the manuscript?
- Have you received reimbursements, fees, funding, or salary from an organization that holds or has applied for patents relating to the content of the manuscript?
- Do you have any other financial competing interests?
- Do you have any non-financial competing interests in relation to this paper?

If you can answer no to all of the above, write 'I declare that I have no competing interests' below. If your reply is yes to any, please give details below.

I declare that I have no competing interests.

I agree to the open peer review policy of the journal. I understand that my name will be included on my report to the authors and, if the manuscript is accepted for publication, my named report including any attachments I upload will be posted on the website along with the authors' responses. I agree for my report to be made available under an Open Access Creative Commons CC-BY license (http://creativecommons.org/licenses/by/4.0/). I understand that any comments which I do not wish to be included in my named report can be included as confidential comments to the editors, which will not be published.

I agree to the open peer review policy of the journal
